# TXNL4B regulates radioresistance by controlling the PRP3‐mediated alternative splicing of FANCI

**DOI:** 10.1002/mco2.258

**Published:** 2023-05-07

**Authors:** Zhao Ju, Jing Xiang, Liang Xiao, Yan He, Le Zhang, Yin Wang, Ridan Lei, Yunfeng Nie, Long Yang, Justyna Miszczyk, Pingkun Zhou, Ruixue Huang

**Affiliations:** ^1^ Department of Occupational and Environmental Health, Xiangya School of Public Health Central South University Changsha Hunan China; ^2^ Department of Radiation Biology, Beijing Key Laboratory for Radiobiology, Beijing Institute of Radiation Medicine AMMS Beijing China; ^3^ Faculty of Naval Medicine Naval Medical University (Second Military Medical University) Shanghai China; ^4^ Department of Ophthalmology, Hunan Clinical Research Center of Ophthalmic Disease, The Second Xiangya Hospital Central South University Changsha Hunan China; ^5^ Xiangya Hospital Central South University Changsha Hunan China; ^6^ Hunan Prevention and Treatment Institute for Occupational Diseases Changsha Changsha Hunan China; ^7^ Department of Experimental Physics of Complex Systems The H. Niewodniczański Institute of Nuclear Physics, Polish Academy of Sciences Kraków Poland

**Keywords:** alternative splicing, ionizing radiation, radioresistance, splicing factors

## Abstract

Ionizing radiation (IR) has been extensively used for cancer therapy, but the radioresistance hinders and undermines the radiotherapy efficacy in clinics greatly. Here, we reported that the spliceosomal protein thioredoxin‐like 4B (TXNL4B) is highly expressed in lung tissues from lung cancer patients with radiotherapy. Lung cancer cells with TXNL4B knockdown illustrate increased sensitivity to IR. Mechanistically, TXNL4B interacts with RNA processing factor 3 (PRP3) and co‐localizes in the nucleus post‐IR. Nuclear localization of PRP3 promotes the alternative splicing of the Fanconi anemia group I protein (FANCI) transcript variants, FANCI‐12 and FANCI‐13. PRP3 regulates alternative splicing of FANCI toward the two variants, FANCI‐12 and FANCI‐13. Radioresistance was greatly enhanced through the combination of PRP31 and PRP8, the critical components of core spliceosome promoted by PRP3. Notably, the inhibition of PRP3 to suppress the production of FANCI‐12 would deprive PRP31 and PRP8 of such interaction. As a result, cell cycle G2/M arrest was induced, DNA damage repair was delayed, and radiosensitivity was improved. Collectively, our study highlights potential novel underlying mechanisms of the involvement of TXNL4B and alternative splicing in radioresistance. The results would benefit potential cancer radiotherapy.

## Graphical abstract

Ionizing radiation (IR) has been extensively used for cancer therapy, but the radioresistance hinders and undermines the radiotherapy efficacy in clinics greatly. Here, we found TXNL4B interacts with PRP3 and co‐localizes in the nucleus post‐IR. Nuclear localization of PRP3 promotes the alternative splicing of the FANCI transcript variants, FANCI‐12 and FANCI‐13, thereby promoting radioresistance. Our study highlights potential novel underlying mechanisms of the involvement of TXNL4B and alternative splicing in radioresistance. The results would benefit potential cancer radiotherapy.

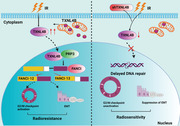

## 1 INTRODUCTION

Lung cancer, which is a leading reason of death and has been reported to have a great prevalence of newly diagnosed about 3 million cases and 2 million deaths only in 2020 globally, only has 18% for 5‐year survival rate in the patient.^1^ Therapy for lung cancer is primarily dependent on cancer staging, history, immunotherapy biomarker testing, and health status. Therapy includes surgical resection, immunotherapy, chemotherapy, and radiotherapy.^2^ Radiotherapy is an important strategy to treat lung cancer, in particular, combined with chemotherapy could improve the efficacy greatly. However, an obstacle to radiotherapy is the development of radioresistance gradually in lung cancer cells. It has shown that the lung cancer cells initially present a dose‐ and time‐response to ionizing radiation (IR), and the resistance may necessitate high doses of radiation to achieve more effective results.^3^, ^4^ The radioresistance declines therapy efficacy, causing severe adverse effects and greatly limiting the life quality. Hence, a deeper appreciation of the molecular mechanisms of radioresistance in lung cancer and more efficient therapeutic targets would be developed, and higher patient survival ratio would be expected.

Amounts of evidence demonstrated that the preferential activation of DNA damage repair, cell cycle checkpoint, and epithelial–mesenchymal transition (EMT) development are typically associated with radiation‐induced resistance.^5^, ^6^ Inhibition of DNA damage repair pathways could enhance the radiosensitivity of cancer cells. A large amount of DNA damage repair–related molecules were developed to be clinical therapy targets, such as p53 and cell cycle checkpoint proteins.^7^, ^8^, ^9^ In addition, the inhibition of EMT development could sensitize the cancer cells to IR.^10^ However, whether there exists other molecular mechanisms remains to be clear.

Alternative splicing, a process of converting the pre‐mRNA to the various mature mRNAs, results in the diversity and complexity of human proteome.^11^ Possible mechanisms of alternative splicing are related to alter protein domains, induce nonsense‐mediated decay, or cause protein truncation.^12^ In fact, in lung cancer, the deregulation of isoforms of splicing regulators exists in common.^13^ Some of the alternative splicing factors have been reported in the regulation of lung cancer. Min Chen et al. found 10 key alternative splicing factors in lung squamous cell carcinoma (LUSC) through bivariate cox regression analysis.^14^ PRP3 (premessenger RNA processing factor 3), one of the noted reported alternative splicing factors, as well as the key component of U4/U6.U5 tri‐snRNA complex, is essential for spliceosome assembly.^15^ Furthermore, PRP3 can regulate a switch of HNF4alpha gene to fetal liver programs in hepatocellular carcinomas^16^ as well as aggravate keratinocyte–derived cutaneous squamous cell carcinoma (cSCCs) severity.^17^ But the function of PRP3 in lung cancer radioresistance is largely unknown.

TXNL4B, the spliceosomal protein thioredoxin‐like 4B, was first reported by Xiaojin Sun et al. It belongs to TXNL4‐family protein serving as a key component of splicing machinery. It is also responsible for the interaction with a series of splice factors such as PRP6 to affect splicing progress through blocking splicing factors’ activity, resulting in pre‐mRNA splicing dysregulation.^18^ Our previous study found that postradiation, TXNL4B expression increased. Further study showed that after knock‐downing TXNL4B expression, lung cancer cells obtained aggravation ability for apoptosis postradiation, which raises the hypothesis that TXNL4B might involve in the regulation of lung cancer radioresistance. Here, we demonstrated that TXNL4B promotes radioresistance in lung cancer cells, whereas the silence of TXNL4B expression reverses the cancer cell resistance status postradiation. TXNL4B binds PRP3 and modulates the latter nuclear translocation postradiation. Mechanistically, PRP3 regulates alternative splicing of FANCI toward the two variants, FANCI‐12 and FANCI‐13. Radioresistance was greatly enhanced through the combination of PRP31 and PRP8, the critical components of core spliceosome promoted by PRP3. Notably, the inhibition of PRP3 to suppress the production of FANCI‐12 would deprive PRP31 and PRP8 of such interactions. As a result, cell cycle G2/M arrest was induced, DNA damage repair was delayed, and radiosensitivity was improved. Collectively, our study highlights potential novel underlying mechanisms of the involvement of TXNL4B and alternative splicing in radioresistance. The results would benefit potential cancer radiotherapy.

## 2 RESULTS

### 2.1 TXNL4B overexpression was found in lung cancer tissues

**FIGURE 1:**
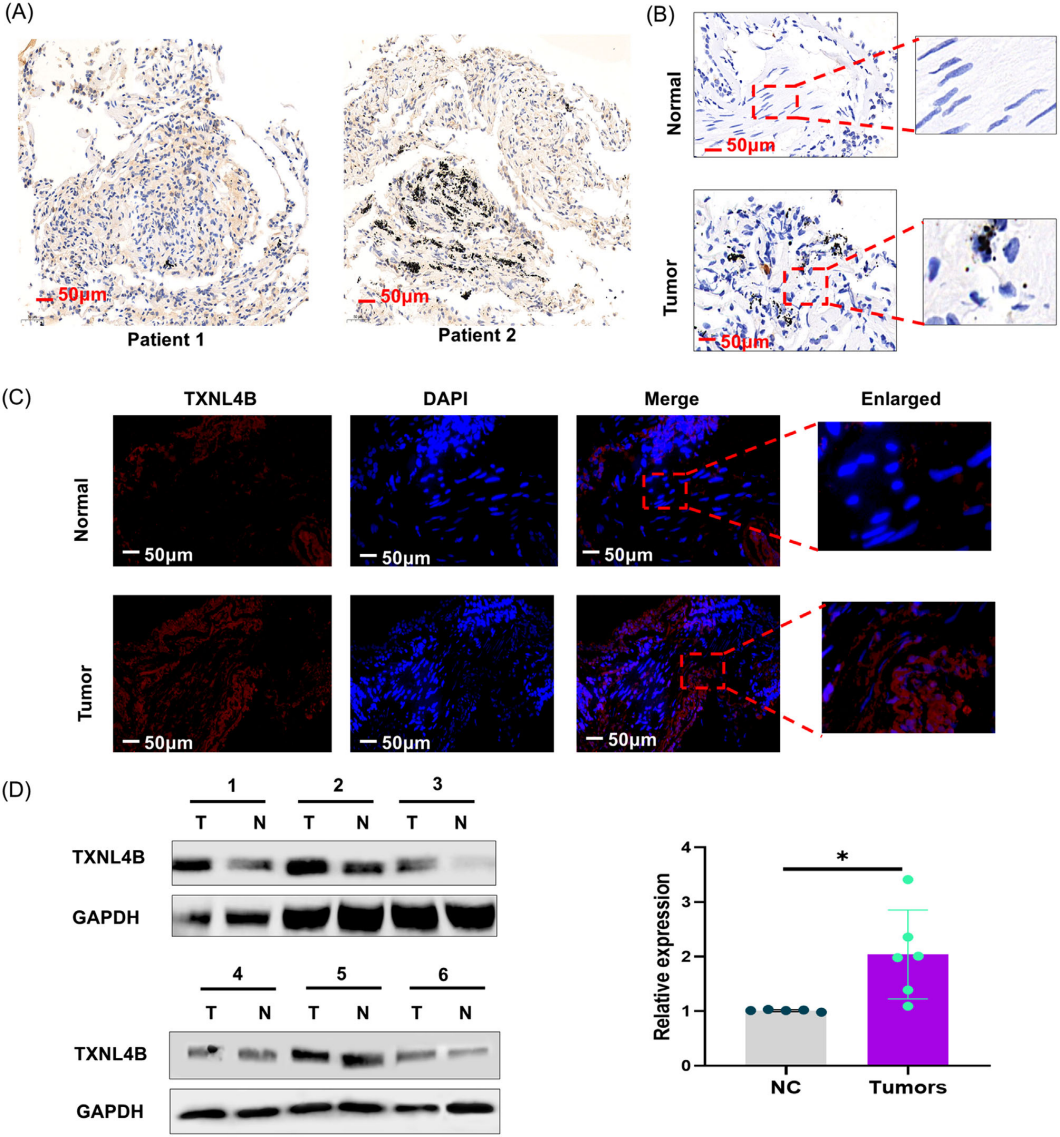
TXNL4B is expressed at high levels in lung tissues: (A) The TXNL4B expression was determined by immunohistochemistry (IHC) in patients who received radiotherapy (*n* = 2). The expression of TXNL4B in lung cancer tissues and adjacent normal tissues from the same patient was assessed by immunohistochemistry (B) (*n* = 4), immunofluorescence (C) (*n* = 4), and Western blotting (D) (*n* = 6). Scale bars, 50 µm. The magnification of the enlarged image is 10 µm.

First of all, lung cancer tissues from patients who received radiotherapy were collected, and the TXNL4B expression was detected. As shown in Figure 1, TXNL4B expression was detected in the lung cancer tissues of patients who received radiotherapy (Figure 1A). Moreover, it showed that TXNL4B expression was higher in cancer tissues than in adjacent normal tissues (Figure 1B). We further revealed that in the cytoplasm of cells, TXNL4B expression was relatively overexpressed compared to the nucleus (Figure 1C). WB detection further confirmed that lung cancer tissues of patients post‐radiotherapy had an overexpression of TXNL4B expression (Figure 1D).

**FIGURE 2:**
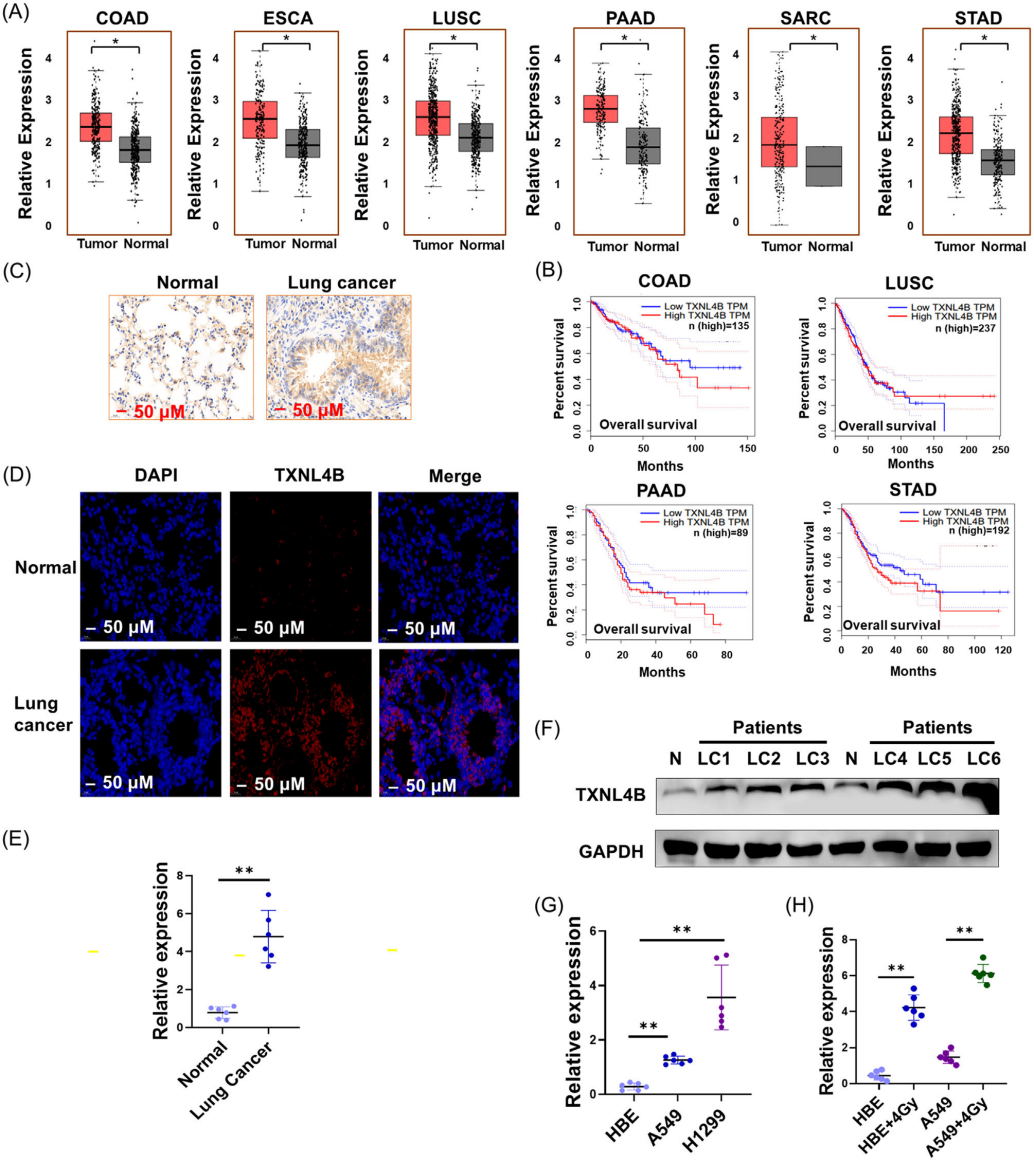
TXNL4B is highly expressed in lung cancer: (A) TXNL4B expression in multiple types of tumor samples and corresponding normal samples from RNA‐seq results of GEPIA (Gene Expression Profiling Interactive Analysis) database (http://gepia.cancer‐pku.cn/detail.php?gene=TXNL4B); (B) Kaplan–Meier curves showing the overall survival of patients of colon adenocarcinoma (COAD), lung squamous cell carcinoma (LUSC), adenocarcinoma of the pancreas (PAAD), and stomach adenocarcinoma (STAD) with low versus high TXNL4B expressions, respectively, *n* = 135, 237, 89, and 192, respectively; (C) the expression of TXNL4B in normal lung tissue, lung cancer (LC) tissues in mice by immunohistochemistry (IHC); (D) representative images of normal lung tissue, LC tissues in mice stained by immunofluorescence (IF); (E) the expression of TXNL4B in normal lung tissues and lung cancer tissues by RT‐PCR, *n* = 6; (F) the expression of TXNL4B in normal lung tissues (N) and lung cancer (LC) tissues by Western blotting; (G) the stability was detected in normal lung cells and various lung cancer cell lines by RT‐PCR; (H) the expression of TXNL4B was detected in HBE (human bronchial epithelial cells) and A549 (human non‐small cell lung cancer cell) post 4 Gy radiation. Nuclei were stained with DAPI (blue). Scale bar = 50 µm. GAPDH was used as positive control. Error bars represent the SD (Standard error). Data are means ± SD from three independent experiments. Two‐tailed, unpaired *t*‐test was used. *p < 0.05, **p < 0.01.

We next evaluated the expression of TXNL4B levels in other various cancers. Using online RNA‐sequencing (RNA‐seq) data from GEPIA (Gene Expression Profiling Interactive Analysis) database (http://gepia.cancer‐pku.cn/), we found TXNL4B expression levels elevated in most of the cancers, including COAD (colon adenocarcinoma), ESCA (esophageal carcinoma), LUSC, PAAD (adenocarcinoma of the pancreas), SARC (sarcoma), STAD (stomach adenocarcinoma) compared with the corresponding adjacent normal tissues (Figure 2A). Survival analysis showed COAD, LUSC, PAAD, and STAD patients accompanied by TXNL4B overexpression presented shorter overall survival duration, whereas patients with lower expression had longer survival duration (Figure 2B). Compared to the normal group, TXNL4B expression was higher in mice with lung cancer (Figure 2C,D). We detected the TXNL4B levels in six patients with lung cancer who received radiotherapy. The expression of TXNL4B protein and mRNA increased both in lung cancer tissues (Figure 2E). We next conducted a retrospective analysis of these six lung cancer (LC) patients with radiotherapy. Four of them (patients of LC3, LC4, LC5, and LC6) had higher TXNL4B expression and presented metastases and recurrences in 12 months, whereas two patients (LC1 and LC2) who had relatively lower TXNL4B expression presented metastases and recurrences in 24 months (Figure 2F).

In lung cancer cell lines, TXNL4B was also overexpressed compared to normal lung cell line (Figure 2G). Using 4 Gy radiation to treat lung cells led to the increased level of TXNL4B (Figure 2H). We next found that TXNL4B level increased in a dose‐ and time‐relationship of radiation in A549 cells (Figure S1A,B). A549 cells are known to be used for the generation of radioresistant cells,^19^ and consistent with our founding that express high levels of TXNL4B. In general, above results inform that TXNL4B may involve in the radioresistant because it was overexpressed in lung cancer patients who received radiotherapy as well as in lung cancer line postradiation.

### 2.2 TXNL4B deficiency is beneficial for radiosensitivity in lung cancer cells

**FIGURE 3:**
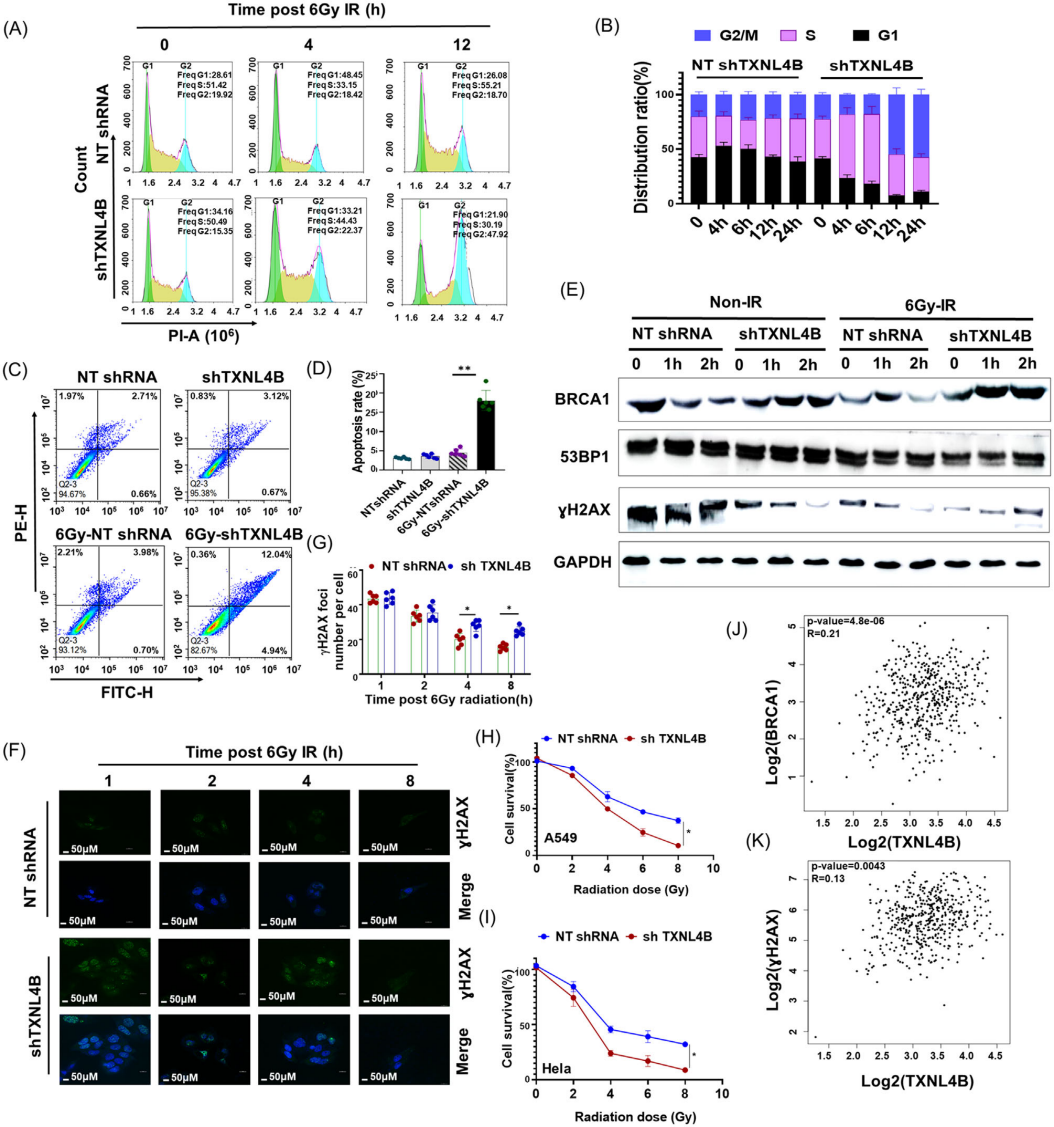
TXNL4B deficiency in lung cancer was positively related to radioresistance: (A) representative images of cell cycle distribution of NT shRNA‐A549 and shTXNL4B‐A549 cells at indicated timepoints post 6 Gy radiation by flow cytometry analysis; (B) quantification analysis cell cycle distribution ratio (%) of NT shRNA‐A549 and shTXNL4B‐A549 cells at indicated timepoints post 6 Gy radiation by flow cytometry analysis; (C) representative images of apoptosis of NT shRNA‐A549 and shTXNL4B‐A549 cells post 6 Gy radiation by Annexin V staining through flow cytometry analysis; (D) quantification analysis of apoptosis of NT shRNA‐A549 and shTXNL4B‐A549 cells post 6 Gy radiation by annexin V staining through flow cytometry analysis; (E) time course showing the total cellular levels of BRCA1, 53BP1, and ɣH2AX protein post 6 Gy radiation in NT shRNA‐A549 and shTXNL4B‐A549 cells by Western blotting assay. GAPDH was subjected to reference control; (F) TXNL4B knockdown decreases the efficiency of DNA double strand break repair as shown by the increased residual ɣH2AX foci post 6 Gy radiation; (G) quantification of ɣH2AX foci. Data are means ± SD from three independent experiments (100 cells for each point were scored in each experiment). Two‐tailed, unpaired *t*‐test. Scale bar, 50 µm. Survival of NT‐shRNA and shTXNL4B of A549 (H) and Hela (I) cells exposed to 6 Gy. Data are means ± SD from three independent experiments. *p < 0.05, **p < 0.01. Correlation of TXNL4B expression and 53BP1 (J) and ɣH2AX (K) in lung cancer tissues based on the RNA‐seq results from GEPIA website (http://gepia.cancer‐pku.cn/index.html).

TXNL4B‐knockdown‐A549 cell line was established stably with shRNA targeting TXNL4B (shTXNL4B) or a non‐targeting shRNA (NT shRNA) as a control. We first found that TXNL4B overexpression or knockdown could influence cell viability in other cancer cells (HepG2 and MDA‐MB‐231) (Figure S2A,B). We next asked whether the TXNL4B could affect the A549 cells’ radioresistant through the modulation of cell cycle, apoptosis, DNA damage repair, cell proliferation, or cell reactive oxygen species (ROS) level. As shown in Figure 3A,B, TXNL4B knockdown influence the cell cycle, leading to an increasing G2/M arrest. At 24 h postradiation, approximately 50% of shTXNL4B cells remained staying in the G2/M phase. Meanwhile, more apoptosis was found in TXNL4B knockdown cells in response to radiation than in the control cells (Figure 3C,D). Compared with non‐radiation, breast cancer susceptibility gene (BRCA1) expression decreased after radiation but increased in the condition of TXNL4B knockout; p53‐binding protein 1 (53BP1) increased after radiation but decreased in the condition of TXNL4B knockout (Figure 3E). TXNL4B knockdown, however, led to gamma H2A histone family member X (γH2AX) foci formed at 4 h postradiation, and the amount of γH2AX maintained to 6 and 8 h postradiation in A549 (Figure 3F). γH2AX foci, a typical DNA repair biomarker, were detected. γH2AX foci‐positive cells did not change in TXNL4B‐knockdown‐A549 cells postradiation at 2 h but decreased gradually up to 6 h postradiation; meanwhile, the expression of γH2AX decreased (Figure 3G). TXNL4B knockdowns affect cell proliferation, making less cell survival percent postradiation (Figure 3H,I). Further analysis exhibited that TXNL4B was positive for BRCA1 and γ. Further analysis exhibited that TXNL4B was positive with BRCA1 andγH2AX expression (Figure 3J,K).

As EMT is crucial for radiotherapy resistance,^20^ we hypothesized the TXNL4B may be associated with EMT regulation and thus promote the radioresistance. Through mice experiment, we further found the structure of lung in mice treated with radiation exhibited alveolar septum widened, patchy fibrosis, but collagen deposition attenuated in the mice with shTXNL4B injection postradiation (Figure S3A). Immunofluorescence (IF) showed vimentin increased after radiation but decreased in the mice lung section after being injected with shTXNL4B (Figure S3B). Transmission electron microscope (TEM) detection showed mitochondrial swelling, matrix clearing, and breakage after radiation but attenuated in the mice with the injection of shTXNL4B after radiation (Figure S3C). We further found that cell cycle–related proteins, including CDK1 expression, increased after radiation but decreased after injected with shTXNL4B prior to radiation, whereas CyclinB expression decreased after radiation but increased after injected with shTXNL4B prior to radiation (Figure S3D). Association analysis showed that TXNL4B was positive for N‐cadherin expression (Figure S3E).

As production and clearance of ROS are associated with radioresistance, and TXNL4B is a mitochondria‐related gene^21^; we thus detected mitochondrial function.

After radiation, the knockdown of TXNL4B resulted in the increased release of ROS level compared to the NT‐shRNA‐A549 cell (Figure S4A). ShTXNL4B‐A549 cells exhibited a reduction in the ATP level compared with NT‐shTXNL4B‐A549 cells postradiation (Figure S4B). In addition, carbonyl cyanide m‐chlorophenyl hydrazone (CCCP) was reduced after radiation in shTXNL4B‐A549 cells in a dose‐dependent fashion (Figure S4C). As it is known that CCCP can trigger the dissipation of the mitochondrial membrane potential leading to ROS release, this result showed reduced CCCP expression in shTXNL4B‐A549 cells after radiation may inhibit the ROS release and oxidative stress progress.^22^ Thus, TXNL4B knockdown in A549 cells enhanced radiosensitivity and was associated with G2/M arrest, DNA damage repair delay, mitochondrial dysfunction, and oxidative stress and EMT process.

### 2.3 TXNL4B is associated with PRP3 nuclear translocation postradiation

**FIGURE 4:**
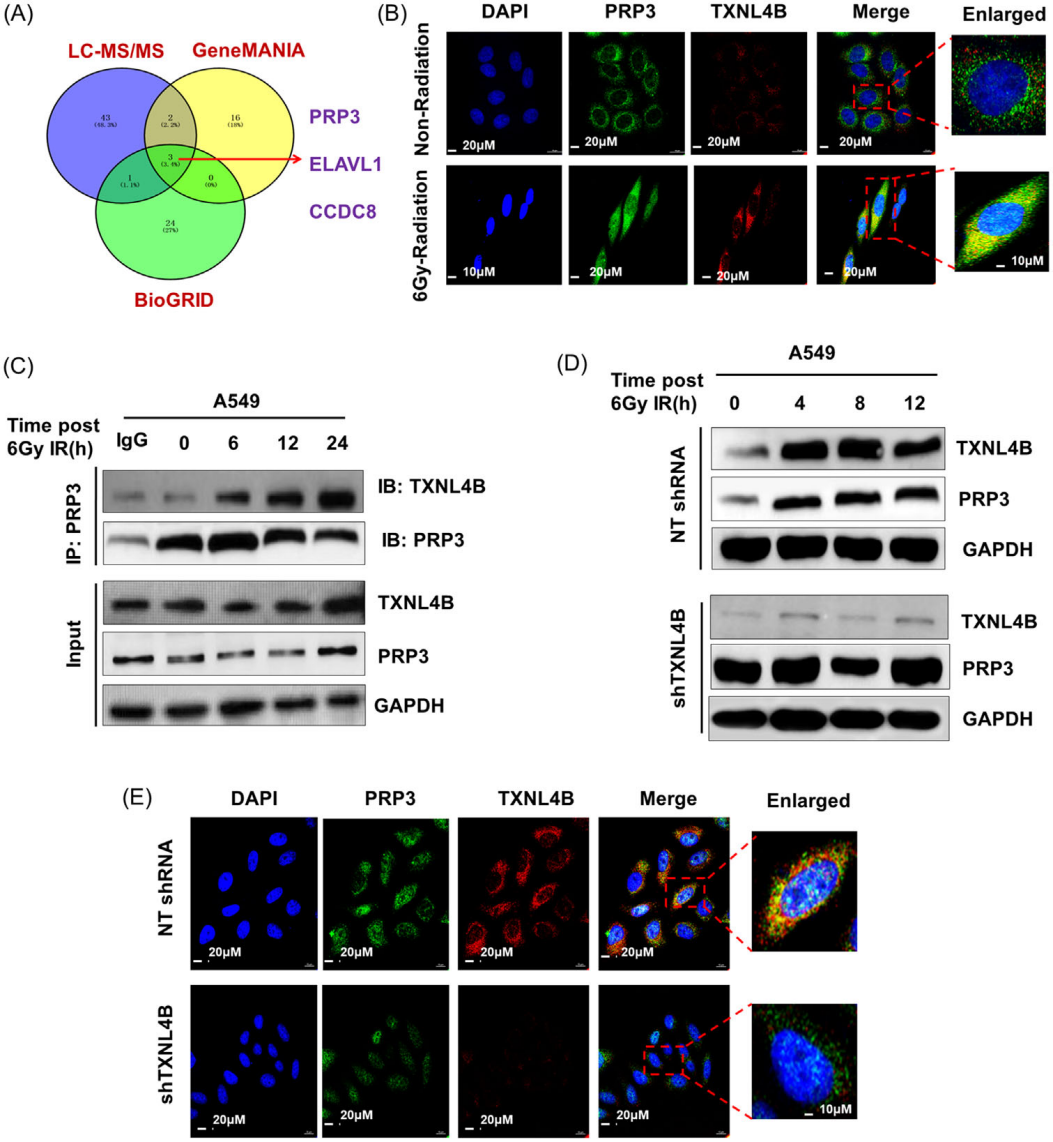
TXNL4B co‐localizes with PRP3 in the nucleus: (A) Venn plot presents the LC–MS/MS technical, GeneMANIA database and BioGRID database used for exploring the overlapping proteins interacted with TXNL4B; (B) confocal microscopy of A549 cells showed TXNL4B co‐localized with PRP3 in the nucleus post 6 Gy radiation; (C) association of TXNL4B with PRP3 was determined by immunoprecipitation (IP) and Western blotting post 6 Gy radiation at indicated timepoints; (D) nuclear proteins from NT‐TXNL4B‐A549 and shTXNL4B‐A549 cells were extracted after radiation, and TXNL4B and PRP3 protein levels were determined by Western blotting; (E) immunofluorescence of NT‐TXNL4B‐A549 and shTXNL4B‐A549 cells after radiation was evaluated using confocal microscopy. Scale bar = 20 µm. The magnification of the enlarged image: scale bar = 10 µm. GAPDH was used as positive control.

Biochemical characterization and crystal structure of TXNL4B were first reported by Simeoni et al. that TXNL4B might be involved in pre‐mRNA splicing.^23^ Further study demonstrated that residues 1–33 of TXNL4B are responsible for their interaction with PRP6,^24^ suggesting TXNL4B is one of the spliceosomal proteins. We hypothesized that TXNL4B could bind to other proteins to form a complex to perform its regulation function postradiation. To test this, we used LC–MS/MS to detect the interaction proteins with TXNL4B; meanwhile, online protein–protein interaction databases (Gene MANIA and BioGRID) were also used to show three proteins, including PRP3, ELAV‐like protein 1 (ELAVL1), and coiled‐coil domain containing 8 (CCDC8), were overlapping interaction proteins with TXNL4B (Figure 4A). PRP3, localized at nuclear (Figure S5A), has been reported as a prognostic biomarker (Figure S5B). PRP knockdown would suppress the cell colony formation (Figure S5C). PRP3 upregulates in the cancer tissues compared with adjacent normal tissues (Figure S5D). Because PRP3 is a splicing factor^25^ and its complex with PRP4 is associated with the U4/U6 small nuclear ribonucleoprotein particle, which is crucial for the activation of spliceosome assembly,^26^ we, therefore, explored the localization of TXNL4B and PRP3 in A549 cells and found some certain co‐localization of both two proteins postradiation (Figure 4B). In addition, it showed that TXNL4B may regulate PRP3 in A549 cells postradiation in a time‐dependent fashion (Figure 4C). As such, we found radiation‐induced upregulation of TXNL4B and PRP3 expression in NT‐shRNA‐A549 cells, but TXNL4B knockdown did not affect the levels of PRP3 expression, suggesting there may exist other mechanisms for the role of TXNL4B in the regulation of PRP3 (Figure 4D).

**FIGURE 5:**
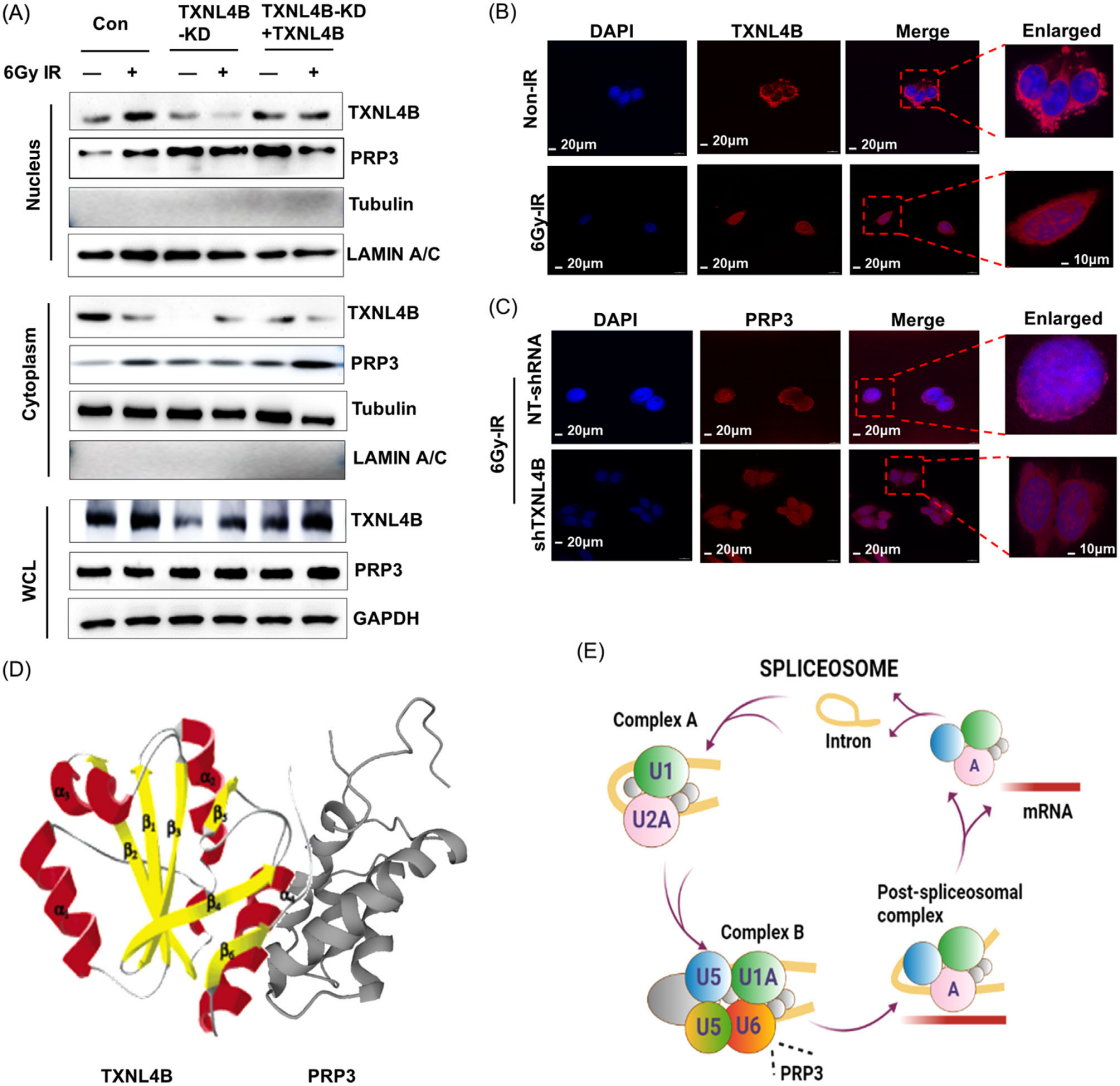
TXNL4B deficiency inhibits the PRP3 translocation from cytoplasm to nucleus: (A) The levels of TXNL4B and PRP3 proteins in the cytoplasm and nucleus, respectively, in TXNL4B‐wt and TXNL4B‐KD and TXNL4B‐knock in A549 cells after 6 Gy irradiation were detected by Western blot analysis. GAPDH served as the internal loading control. Tubulin and LAMIN A/C proteins were detected as the control cytoplasmic and nuclear proteins, respectively. Exogenous TXNL4B‐expressing vectors were transfected into TXNL4B‐KD cells for the rescue experiment; (B) immunofluorescence of TXNL4B cell location after 6 Gy radiation was evaluated using confocal microscopy. Scale bar = 20 µm. The magnification of the enlarged image: scale bar = 10 µm; (C) immunofluorescence of PRP3 cell location NT‐TXNL4B‐A549 and shTXNL4B‐A549 cells after 6 Gy radiation was evaluated using confocal microscopy. Scale bar = 20 µm. The magnification of the enlarged image: scale bar = 10 µm; (D) 3D protein structure of TXNL4B^23^ and PRP3 (available online Uniprot database); (E) procedure of spliceosome (available online KEGG).

As many genes, participating in splicing and transcription, are translocation partners for translocation regulation,^27^ we postulated that TXNL4B may modulate the nuclear translocation of PRP3 postradiation. The distributions of TXNL4B and PRP3 in cell cytoplasm and nucleus postradiation were detected. After radiation, TXNL4B moved into nuclear in order to co‐localize with PRP3 in NT‐RNA‐A549 cells. Conversely, TXNL4B knockdown hindered the nuclear translocation of PRP3 postradiation (Figure 4E). Both TXNL4B and PRP3 expressions were increased in the nucleus postradiation, but TXNL4B knockdown declined the nuclear translocation of PRP3 as its nuclear level decreased (Figure 5A–C). Because PRP3 is a key splicing factor, our results indicated that TXNL4B may enhance splicing factor translocation from cytoplasm to the nuclear, which raises the hypothesis that TXNL4B might regulate the function of the splicing factor in alternative splicing through nuclear translocation in response to radiation.

### 2.4 PRP3 deficiency affects the alternative splicing of radiation‐related genes

As shown in Figure 5D, the 3D structure of TXNL4B and PRP3 can bind together potential, as well as PRP3 plays essential roles in the spliceosome pre‐mRNA splicing (Figure 5E), whether PRP3 is involved in the regulation of alternative splicing in lung cancer cells postradiation needed to be evaluated. Splicing events regulated by PRP3 either in its wild type or knockdown status were evaluated by the multivariate analysis of transcript splicing after performing RNA‐seq detection (Figure S6A). Kyoto Encyclopedia of Genes and Genomes (KEGG) analysis showed that top 30 changed genes were enriched in the signaling transduction and cellular community pathways (Figure S6B). Differential alternative splicing frequency showed most of the genes present exon skipping (SE) (Figure S6C). Scatter plot showed the number of alternative splicing events that occurred in upregulation and downregulation genes (Figure S6D). We further validated some gene expressions by qRT‐PCR. It showed that the genes involved in spliceosome pre‐mRNA splicing, such as floral binding protein 11 (FBP11) and pre‐mRNA‐splicing factor 22 (RBM22), changed significantly after PRP3 knockdown (Figure S6E). Sashimi plot illustrated the reads crossing exon skipping junctions that occurred in stable PRP3‐knockdown cells postradiation (Figure S7). It showed PRP3 knockdown markedly led to spliceosome pre‐mRNA splicing‐related genes to various variants, for example, the LYRM (LYR motif containing) 1‐1 (Figure S7A), the LYRM1‐2 (Figure S7B), the PTPN22 (protein tyrosine phosphatase non‐receptor type 22)‐4 (Figure S7C), PTPN22‐5 (Figure S7D), ERCC1 (excision repair cross‐complementation group 1)‐10 (Figure S7E), and OBSL1 (obscurin like cytoskeletal adaptor 1)‐17 (Figure S7F). These results indicated that PRP3 knockdown would change the alternative splicing of some genes in A549 cells in response to radiation.

**FIGURE 6:**
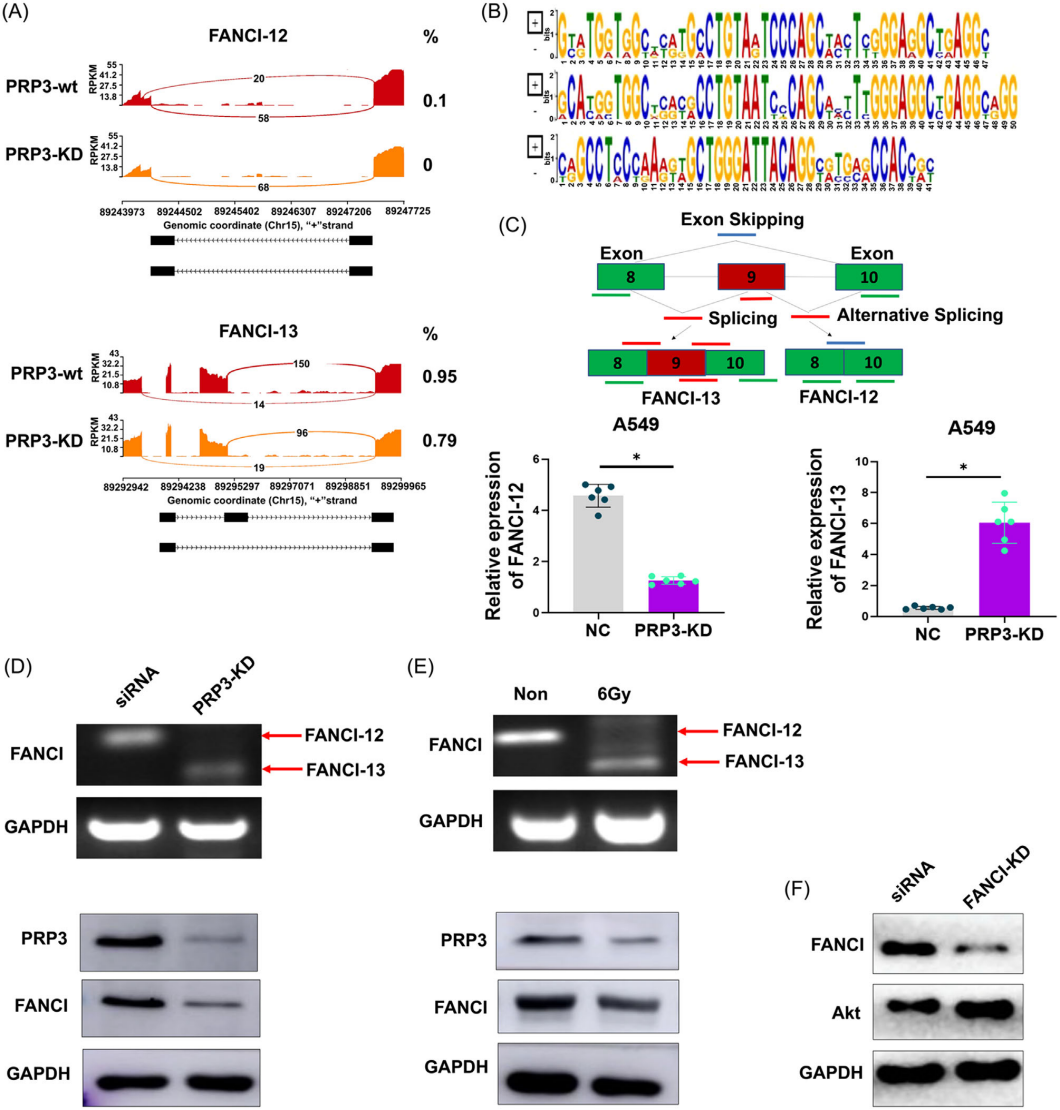
PRP3 is necessary for FANCI pre‐mRNA and full expression of FANCI protein; (A) Sashimi plots of exon skipping reads of FANCI alternative splicing in PRP3‐wt or PRP3‐KD cells. Red box highlights the location of exon skipping in the FANCI mRNA; (B) identification of FANCI‐binding motif in the exon skipping mRNA region by MEME motif analysis (https://meme‐suite.org/meme/); (C) expression analysis of two FANCI alternative splicing variants, FANCI‐12 and FANCI‐13, after PRP3 knockdown in A549 cells; (D) RT‐PCR analysis and Western blotting analysis of FANCI, FANCI‐12, FANCI‐13, and PRP3 expressions after PRP3 knockdown in A549 cells; (E) RT‐PCR analysis and Western blotting analysis of FANCI, FANCI‐12, FANCI‐13, and PRP3 expressions after 6 Gy radiation in A549 cells; (F) expression of FANCI and Akt were analyzed after FANCI knockdown by Western blotting in A549 cells. Data are means ± SD from three independent experiments. Two‐tailed, unpaired *t*‐test was used. *p < 0.05.

Among these candidate genes (Table S1 and Table S2), we focused on FANCI, in which one of the exons was skipped after PRP3 knockdown postradiation and two variants FANCI‐12 and FANCI‐13 have been present (Figure 6A). MEME motif analysis (http://meme‐suite.org/) showed the PRP3‐binding motif in the exon skipping is coding mRNA region due to there exists enrichment of nucleotides G and A (Figure 6B). FANCI, reported as potential with carcinogenesis,^28^ is associated with DNA damage repair pathway and cancer sensitization with chemo‐ or radiotherapy.^29^ We speculated that PRP3 deficiency may involve in the mediation of FANCI alternative splicing occurrence. RT‐PCR was conducted to detect the splice variants of FANCI mRNA to verify the RNA‐seq result (Figure 6C), and the results were consistent. It was also revealed that PRP3 knockdown (Figure 6D) and 6 Gy radiation (Figure 6E) inhibited FANCI‐12 expression but promoted FANCI‐13 expression (Figure 6D,E). PRP3 knockdown also inhibited FANCI expression either treated or without treated radiation. We further silenced FANCI expression and found the Akt expression, which is a key regulation in ROS increased (Figure 6F). These results showed that PRP3 knockdown causes a switch of the FANCI splicing isoform from FANCI‐12 to FANCI‐13 during the response to radiation.

### 2.5 FANCI isoforms differentially regulate cell cycle checkpoints and EMT

**FIGURE 7:**
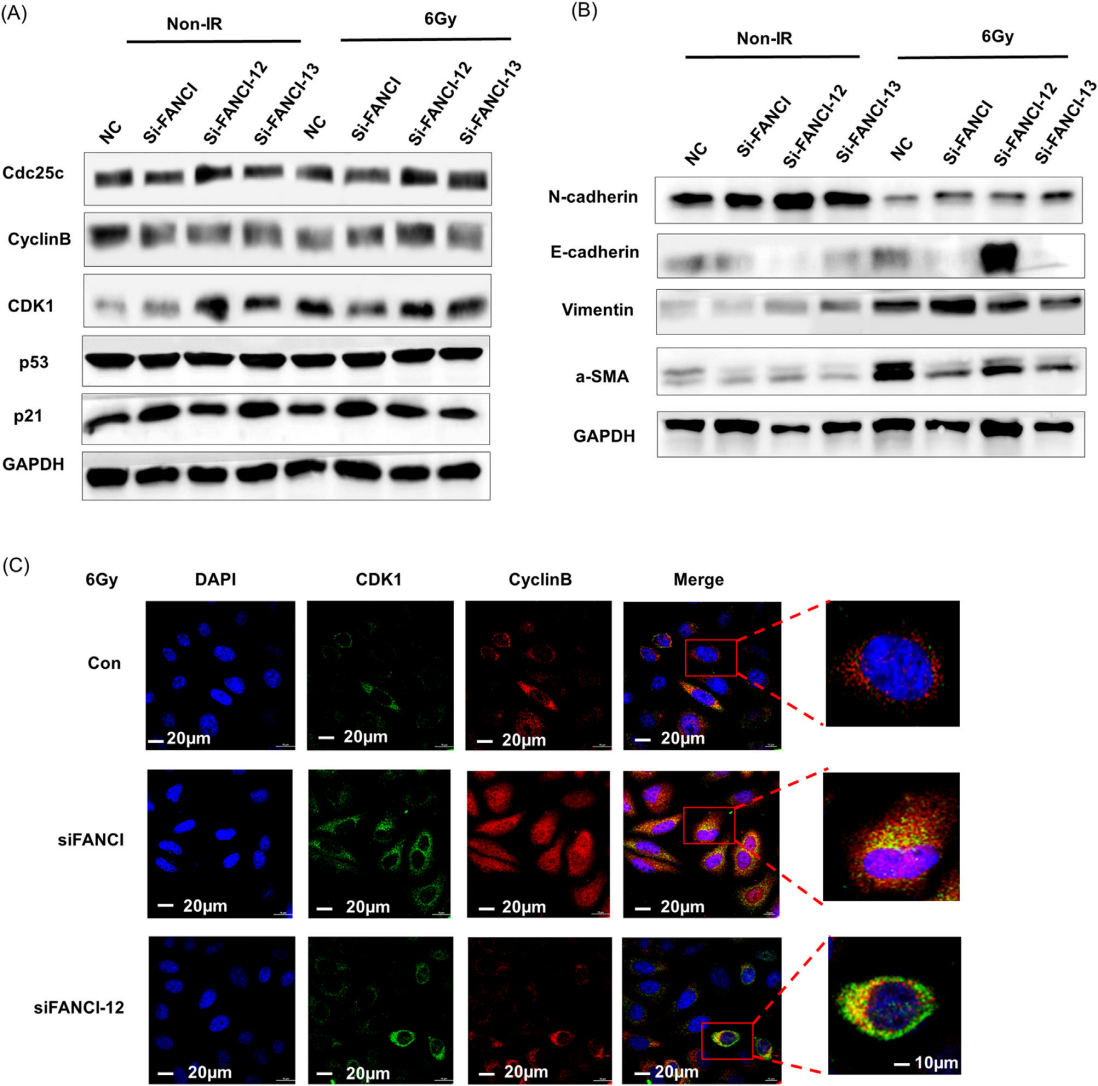
FANCI isoforms differential regulate cellular radiation‐induced cell cycle checkpoint proteins and epithelial–mesenchymal transition (EMT)‐related proteins: (A) Cell cycle checkpoint‐related proteins were determined after knockdown FANCI and FANCI variants post 6 Gy radiation by Western blotting. GAPDH serves as control; (B) EMT‐related proteins were determined after knockdown FANCI and FANCI variants post 6 Gy radiation by Western blotting. GAPDH serves as control; (C) A549 cells with stable reduction of FANCI or control were transiently transfected with siRNA‐FANCI‐12, to determine the co‐location of CDK1 and CyclinB post 6 Gy radiation by confocal microscope. Scale bar = 20 µm. The magnification of the enlarged image: scale bar = 10 µm.

Next, whether two FANCI isoforms, FANCI‐12 and FANCI‐13, have functions in the regulation of radiation‐induced cell damage was evaluated. To this end, we knocked down the FANCI, FANCI‐12, and FANCI‐13 expressions in A549 cells, respectively. We first observed that FANCI‐12 knockdown led to the inhibition of cell proliferation postradiation compared with siNC and radiation (Figure S8A), whereas FANCI‐13 knockdown did not affect cell proliferation postradiation (Figure S8B). We then used Western blotting to detect cell cycle–related checkpoints. We found FANCI‐12 knockdown increased cell division cycle 25C (cdc25c), CyclinB, and Cyclin‐dependent kinase 1 (CDK1) expression postradiation compared with siNC radiation treatment in A549 cells, but FANCI and FANCI‐13 knockdown did not change the expression levels of cdc25c, CyclinB, and CDK1 (Figure 7A). In addition, we found that FANCI‐12 knockdown increased E‐cadherin expression and decreased N‐cadherin, vimentin, and alpha smooth muscle actin (α‐SMA) expression levels postradiation compared with siNC radiation treatment in A549 cells, but FANCI and FANCI‐13 knockdown did not change the expression levels of cdc25c, CyclinB, and CDK1 (Figure 7B). We then asked whether an overexpression of FANCI‐12 or FANCI‐13 can affect the DNA damage repair and EMT.

**FIGURE 8:**
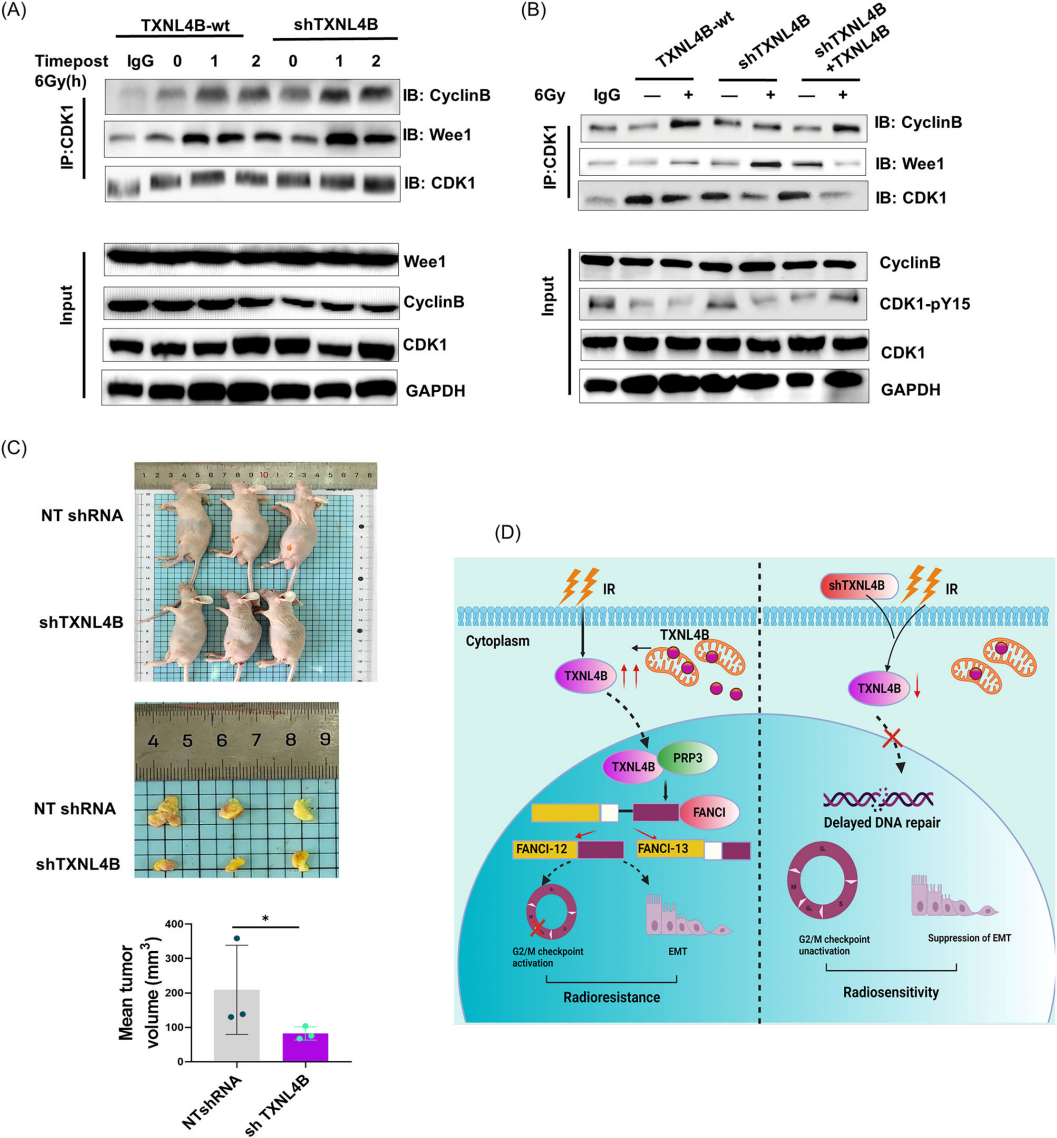
TXNL4B knockdown synergizes with radiotherapy in a xenograft model of lung cancer: (A) associations of CDK1 with Cyclin B and Wee1 were determined by immunoprecipitation (IP) and Western blotting post 6 Gy radiation at indicated timepoints in TXNL4B‐wt and shTXNL4B cells. GAPDH serves as control; (B) associations of CDK1 with Cyclin B and Wee1 were determined by immunoprecipitation (IP) and Western blotting post 6 Gy radiation in TXNL4B‐wt and shTXNL4B cells and TXNL4B knock in cells. GAPDH serves as control; (C) tumors in the NT shRNA and shTXNL4B groups 35‐day postradiation treatment and the representative images and quantitative results of mean tumor volumes (mm^3^) in two groups. *n* = 3 each group; (D) model of suggested regulation of TXNL4B roles in the radioresistance in lung cancer. In response to radiation, TXNL4B upregulation is associated with the interaction of splice factor PRP3, which ultimately activates FANCI alternative splicing variant FANCI‐12, resulting in the CDK1 and Cyclin B interaction to promote the radioresistance. TXNL4B knockdown prevents interaction with PRP3 postradiation, subsequent delays of G2/M checkpoint activation, and epithelial–mesenchymal transition (EMT) procedure lead to radiosensitivity.

As an increased level of cdc25c can promote the interaction of CDK1 and CyclinB to phosphate CDK1, leading to G2/M arrest,^8^ we, therefore, explored the localization of CDK1 and CyclinB in A549 cells and found after radiation. We observed the colocalization of aggregated CDK1 and CyclinB in the nucleus in FANCI‐12‐knockdown‐A549 cells compared with siNC postradiation (Figure 7C). Moreover, we also found TXNL4B knockdown can promote increased expression of cdc25c, leading to the increased interaction of CDK1 and CyclinB through Co‐IP and Western blotting (Figure 8A,B). These results indicated that PRP3 inhibits radioresistance at least in part due to promoting the production of FANCI‐12.

### 2.6 PRP3 regulates FANCI alternative splicing by modulating the interaction between PRP31 and PRP8

As PRP3 involves in the spliceosome complex B (U4/U6/U5/U1), we hypothesized that PRP3 dysfunction may cause the alteration of other proteins’ interaction, which results in the cellular radioresistance. PRP3 promotes the complex B (PRP18, PRP19, and PRP8) expression in germline stem cells^30^ and promotes the interaction of PRP31 and PRP4,^31^ which leads to the stabilization of complex B. We, therefore, hypothesized that the disassembly of complex B causes a switch of FANCI alternative splicing isoforms from FANCI‐12 to FANCI‐13. To test this, we first evaluated the expression of PRP31 and PRP8 in PRP3 knockdown or postradiation. Importantly, PRP31 and PRP8 were downregulated postradiation in PRP3‐knockdown‐A549 cells (Figure S9A). Intriguingly, as observed in PRP3‐knockdown cells, knockdown of PRP31 or PRP8 led to a decrease in the level of FANCI protein and FANCI mRNA exon skipping, and the PRP31 protein expression also decreased (Figure S9B). These observations indicated that PRP3 regulates FANCI alternative splicing by modulating the interaction between PRP31 and PRP8, the key components of complex B. Considering that PRP31 and PRP8 were downregulated in cells postradiation, these findings also demonstrate that PRP3 plays a functional role in the early step of cellular response to radiation.

### 2.7 TXNL4B promotes radioresistance of lung cancer in vivo

Above results indicated that the upregulation of TXNL4B aggravated resistance to radiation from cell experiment's perspective. Then, we further identified TXNL4B function from animal experiment's perspective. Nude mice were divided into two groups, one injected with NT‐shRNA‐A549 cells to establish lung cancers, whereas another group injected with TXNL4B‐knockdown cells. While the induced volumes approached near 200 mm^3^, a single 6 Gy dose of radiation was intervened. After 35 days later,the tumor volumes were compared between two groups. We found that, compared with the control group, mice treated with the combination of radiation and TXNL4B knockdown had a trend of tumor regression, whereas, in control group, the tumors kept on growing (Figure 8C).

## 3 DISCUSSION

Converging evidence demonstrates that cell cycle, EMT, and DNA damage repair are hallmarks of cancer cells in response to radiation,^7^, ^9^ and importantly, alternative splicing has been identified to play a vital role in regulating cancer development and progression and resistance.^32^ However, how the alternative splicing controls the cancer cells’ action in response to radiation is still incompletely clear. Here we show that, in A549 cells, TXNL4B overexpression is associated with PRP3 nuclear translocation, and PRP3 knockdown causes FANCI alternative splicing after radiation. We suggest that the PRP3, serving as a splicing factor, can be modified by TXNL4B, resulting in promoting G2/M transition, EMT, and DNA repair and contributing to the observed radioresistance of lung cancer through the regulation of FANCI splicing isoforms. Our findings indicate a mechanistic model for the regulation of PRP3 by TXNL4B and the regulation of FANCI splicing isoforms by PRP3 in lung cancer that may have consequences for the susceptibility of this cancer type to radiotherapy (Figure 8D).

Many factors have been indicated to play roles in radioresistance. Cell cycle, DNA damage repair, and EMT affect response to radiotherapy.^33^ TXNL4B, also named Dim2 previously, is a paralogue of Dim1.^24^ TXNL4B has been shown to be required for S/G2 transition in cell cycle.^18^ Mamo et al. showed that in vitro matured bovine oocytes, TXNL4B, were overexpressed and linked with cell cycle regulation through transcript sequence analysis.^34^ Till now, very little is known about the function of TXNL4B in the regulation of cellular response to radiation. TXNL4B falls into our attention due to our previous work that it has increased expression in A549 cells after radiation through RNA‐seq analysis.^35^ Through investigation in this study, we found a clear and interesting regulation of TXNL4B in cell cycle, EMT, and DNA damage repair. These observations indicate that an overexpression of TXNL4B postradiation may facilitate G2/M checkpoint activation and DNA repair and EMT.

PRP3, small nuclear ribonucleoprotein U5 subunit 200, is a splicing factor that is the constituent of the U4/U6.U5 tri‐snRNP complex and is ubiquitously expressed in all tissues.^36^ Previous study has reported that PRP3 overexpression in cSCCs was associated with poor prognosis in cSCCs patients.^17^ The SUMOylation of PRP3 is required for U4/U6.U5 tri‐snRNP complex formation, suggesting PRP3 regulation is a critical hallmark in governing spliceosome assembly and affects the splicing efficiency.^37^ Another modification such as ubiquitination and de‐ubiquitination of PRP3 affects its interaction with PRP8.^38^ Nuclear translocation is another type of regulation of protein activity.^39^ For example, SRSF3, another splicing factor, is phosphorylated and led to its nuclear translocation in the regulation of facial mesenchyme.^40^ Our results showed PRP3 nuclear translocation postradiation occurred followed by TXNL4B and PRP3 complex formation postradiation. Conversely, the suppression of TXNL4B would disrupt the PRP3 nuclear translocation postradiation resulting in hindered G2/M checkpoint activation, DNA repair, and EMT progression. Based on these findings, it would be potential for developing precise and effective radiotherapy targets in lung cancer cells through blocking TXNL4B‐mediated PRP3 activity and translocation in clinics.

Previously, FANCI has been shown to associate with carcinogenesis, which knock down the FANCI expression promotes DNA damage and makes ovarian cancer cells more sensitive to chemotherapy.^29^ A study analyzed the FANCE splice isoform and found this splice variant is high expressed in breast cancer individuals, and this isoform can be efficiently translated into a functional protein to block into G2/M phase, leading to the reduction of cell survival.^41^ A study analyzed FANCA gene in breast cancer patients and found multiple alternative splicing events, including the insertion of intronic portion and exon skipping, suggesting the alternative splicing events of FANCA may play critical function in biology.^42^ Online TCGA splice‐seq database (Cancer Splicing Visualized) illustrated that in lung cancer patients, most of FANCI alternative splicing events are exon skipping, for instance, the PSI (possible splicing index) of exon 34 skipping is almost 98.7% incidence (https://bioinformatics.mdanderson.org/TCGASpliceSeq/singlegene.jsp), indicating these splicing events may involve in the regulation of lung cancer development and process. Here, in our study, after radiation, alternative splicing results in two transcript variants, FANCI‐12 and FANCI‐13 in PRP3‐knockdown‐A549 cells. FANCI‐12 is stable and performs function, but FANCI‐13 is unstable. Meanwhile, the splicing factor PRP3 is necessary for the generation of FANCI variants in A549 cells postradiation. PRP3 occurred during nuclear translocation postradiation in TXNL4B‐knockdown cells and was downregulated postradiation; this indicated that PRP3 knockdown would induce the alternative splicing of FANCI. In addition, we found that PRP3 knockdown weakened the interaction of PRP31 and PRP8, which are key components for the assembly of U4/U6.U5 tri‐snRNP; this, in turn, led to FANCI exon skipping. As reported, the stability of the U4/U6.U5 tri‐snRNP is mainly dependent on the interaction of splicing factors such as PRP31 and PRP8.^43^ Consistent with these previous reports that we confirmed that PRP3 stabilized the U4/U6.U5 tri‐snRNP through regulation of interaction within PRP31 and PRP8. More importantly, PRP3 knockdown led to the downregulation of PRP31 and PRP8 in the A549 cells after radiation, suggesting PRP8 and PRP31 may be other targets of radioresistance. This further identified that PRP3 is a critical regulator of radiation‐induced radioresistance. In summary, our data demonstrate that serving as a splicing factor, PRP3 can regulate its downstream targets leading to the radioresistance‐related genes dysfunction, but PRP3 itself can be regulated by upstream TXNL4B, which can interact with PRP3 to influence the unclear translocation of PRP3.

Actually, approximately 90% of genes may encounter alternative splicing, which is a way to generate protein diversity for human evolution.^44^ Here, in our study, we aimed to investigate the role of the TXNL4B‐PRP3‐FANCI axis in the radioresistance of lung cancer cells. Radioresistance is a multistep and highly dynamic process as well as regulated by multiple factors. Previous reports have found that a couple of regulators related to radioresistance produced various isoforms through being spliced, presenting totally different functions. For example, somatostatin receptor splicing variant *sst*5TMD4 overexpression is associated with poor survival and increased resistance to radiotherapy/chemotherapy.^45^ Alternative splicing of coactivator‐associated arginine methyltransferase 1 (CARM1) by epithelial splicing regulatory protein 1 (ESRP1) resulted in lung cancer cells sensitize to chemotherapy.^46^ STAT3β, one of the spliced isoforms of (Signal Transducer and Activator of Transcription 3) STAT3, serving as a dominant‐negative regulator, enhances sensitivity to radiotherapy^47^ but STAT3 itself attenuates the radiosensitivity on the contrary.^48^ Our study found that the alternative splicing of FANCI would produce two isoforms, of which one was showed aggravating lung cancer cells’ radioresistance, whereas another had no effects. Previous studies and our study demonstrate that while taking measures for the prevention and treatment of radioresistance, it should be paid more attention to the effects of alternative splice, which might offer a new and challenging approach to reverse therapeutic resistance.

Using A549 cell line for functional and mechanistic studies was a limitation in this study, which may attenuate the reliability of the conclusion; future studies will be conducted in multiple lung cancer cell lines. Another limitation is that the proposed mechanism was not confirmed in mice xenograft models that needed further investigation. In summary, our data demonstrate that TXNL4B is upregulated after radiation and is associated with radioresistance through the regulation of G2/M checkpoint activation, the promotion of DNA repair, or the EMT process. TXNL4B knockdown causes PRP3 nuclear translocation. The PRP3 knockdown resulted in the alternative splicing of FANCI. One of FANCI transcript variants, FANCI‐12 knockdown may promote G2/M arrest and apoptosis. Mechanistically, PRP3 knockdown leads to the unstabilized U4/U6.U5 tri‐snRNP postradiation though affecting the physical interaction of PRP31 and PRP8. Our studies provide novel insights regarding the possibility of RNA splicing machinery working with cellular radioresistance. Notably, the modulation of RNA splicing may be a challenging and promising approach for improving the radiotherapy resistance.

## 4 MATERIALS AND METHODS

### 4.1 Patient samples

Lung fresh tissues and adjacent normal tissues of 10 patients were recruited from September 2021 to February 2022 at the Oncology Department of Xiangya Hospital, Central South University, and Hunan Province Occupational Disease Prevention and Control Hospital, Changsha, China. Paraffin‐embedded archived lung cancer tissues used for hematoxylin and eosin staining, immunohistochemistry (IHC), and IF analysis were collected from five patients at the Department of Pathology, the Second Affiliated Hospital, Central South University from September 2021 to February 2022. All included patients provided informed consent, and the Ethics Committee of the School of Public Health, Central South University approved this study (Approval No. XYGW‐2018‐08). The lung cancer was diagnosed based on the clinical‐related criteria.^49^ The inclusion criteria of samples from lung cancer patients who received radiotherapy were (i) received thoracic radiotherapy (RT) for lung cancer, (ii) were aged over 20 years, and (iii) provided written informed consent. Exclusion criteria: (i) companied by other lung diseases, including pulmonary tuberculosis (TB); (ii) companied by other cancers; (iii) refused to accept the informed consent.

### 4.2 Cell culture and cell treatments

The human lung cancer cell lines A549, H1299, and HBE, purchased from the American Type Culture Collection (ATCC, Manassas, VA, USA), are stored in our lab. The Hela, MDA‐MB‐231, and HepG2 cell lines, purchased from the Cell Bank of Type Culture Collection of the Chinese Academy of Sciences (Shanghai, China), are stored in our lab as well. These cell lines were cultured according to the instructions. In brief, Dulbecco's modified Eagle's medium (DMEM; HyClone, Utah, USA) with 10% fetal bovine serum and 1% antibiotic in a 5% CO_2_ cell incubator at 37°C were selected. Lentiviral vectors expressing shRNA against TXNL4B, a control non‐targeting (NT) shRNA, or a full‐length sequence of TXNL4B, were transfected into the cells, and the cells were selected by the addition of puromycin. TXNL4B knockdown and overexpression were confirmed by three real‐time PCR analyses and Western blotting. All transient transfections were performed using Lipofectamine 2000 (Invitrogen) according to the manufacturer's instructions. All cell irradiation treatments were performed using a Co‐60 source; the dose rate of the system was 0.69 Gy/min. The radiation resource was provided by Beijing Key Laboratory for Radiobiology, Beijing Institute of Radiation Medicine, AMMS, Beijing, China.

### 4.3 TXNL4B knockdown and overexpression

For TXNL4B knockdown, the following shRNA oligonucleotides were used as the target sequences for TXNL4B knockdown in the cancer cell lines: shRNA TXNL4B5′‐GCTTCCTACTGCCCAAGCT−3′ and non‐targeting (NT): 5′‐AATAAGGGCAATACC CCA G‐3′. The shRNAs were cloned into a pGreen puro shRNA vector (SystemBio) to obtain pGreen puro‐shTXNL4B and pGreenpuro‐NT shRNA *Lentivirus* vectors. For an overexpression of TXNL4B, the lentiviral vector GV367 containing the full‐length sequence of human TXNL4B was constructed by Shanghai GenePharma Co., Ltd (Shanghai, China). These vectors were then transfected into the HEK‐293T cell line.

### 4.4 RNA interference targeting sequences

siRNAs listed in this study were synthesized by GenePharma Co., Ltd (Shanghai, China). Briefly, while performing siRNA transfection, Lipofectamine 2000 (Invitrogen) was used, and the transfection steps followed the instructions introduced by the company. The sequences of siRNAs were as follows:

TXNL4B‐siRNA, GCUUCCUACUGCCCAAGCU;

PRP3‐siRNA, GGAGUAAAGAAGCGACGAAUATT;

PRP6‐siRNA, GGUGGACUAAACACUCCAUACTT;

FANCI‐siRNA, GCAAGGAGAUUCCAAUAAUTT;

FANCI‐12‐siRNA, GCCGAGUUCCAGAUGGAAACATT;

FANCI‐13‐siRNA, GAAGCCACUGAAGAGCUUUAATT.

### 4.5 Cell colony assay

Briefly, A549 cells were cultured in 60‐mm dishes. Then the indicated dose of radiation was taken to expose cells. After radiation, cells were incubated in a 4‐mL medium and cultured for 1 week to allow colony formation. Cells were stained with 0.5% crystal violet in phosphate‐buffered saline (PBS) with 20% methanol, and colonies with >50 cells were counted.

### 4.6 Antibodies and constructs

The antibodies used in this study were anti‐TXNL4B (12927‐1‐AP; 1:1000 for Western blotting; Proteintech), anti‐E cadherin (3195s;1:1000 for Western blotting and 1:200 for immunofluorescence; Cell Signaling Technology), anti‐N‐Cadherin (13116s; 1:1000 for Western blotting and 1:200 for immunofluorescence; Cell Signaling Technology), anti‐Vimentin (5741S; 1:1000 for Western blotting and 1:200 for immunofluorescence; Cell Signaling Technology), anti‐CDC8 (sc‐365925; 1:1000 for Western blotting and 1:200 for immunofluorescence; Santa Cruz Biotechnology), anti‐Akt (4691S; 1:1000 for Western blotting and 1:400 for immunofluorescence; Cell Signaling Technology), anti‐ɣH2AX (05‐636; 1:1000 for Western blotting and 1:500 for immunofluorescence; Millipore), anti‐cdc25c (sc‐13138; 1:1000 for Western blotting and 1:500 for immunofluorescence; Santa Cruz Biotechnology), anti‐CyclinB (sc‐166210; 1:1000 for Western blotting and 1:500 for immunofluorescence; Santa Cruz Biotechnology), anti‐cdk1 (9116S; 1:1000 for Western blotting and 1:500 for immunofluorescence; Cell Signaling Technology), anti‐p53 (2527S; 1:1000 for Western blotting and 1:500 for immunofluorescence; Cell Signaling Technology), anti‐p21 (2947S; 1:1000 for Western blotting and 1:500 for immunofluorescence; Cell Signaling Technology), anti‐α‐SMA (19245S; 1:1000 for Western blotting and 1:200 for immunofluorescence; Cell Signaling Technology), anti‐wee1 (sc‐5285; 1:1000 for Western blotting and 1:500 for immunofluorescence; Santa Cruz Biotechnology), anti‐cdk1‐pY15 (4539S; 1:1000 for Western blotting and 1:50 for immunofluorescence; Cell Signaling Technology). PRP3 (cat. no. # ab50386, Abcam).

### 4.7 EdU assay

To detect DNA synthesis after IR, the thymidine analog EdU was used as a probe. The cells were seeded on confocal dishes at 1 × 10^5^ cells/dish and cultured to reach a normal growth state. After the cells were treated, a BeyoClickTM EDU Cell Proliferation Kit with Alexa Fluor 488 (Beyotime) was used to detect DNA that carried integration of the synthetic nucleotide EdU. The cells were observed by X‐Light V3 (Crest Optics) confocal microscopy. EdU‐positive cells were counted using ImageJ (National Institutes of Health).

### 4.8 Immunofluorescence (IF) and immunohistochemistry (IHC) analyses

IF and IHC detection were performed as previous report. In brief, for IF, the steps were that after fixation with 4% paraformaldehyde, cultured cells and frozen sections of lung tissues were cleared with 0.3% Triton X‐100 and blocked in 2% bovine serum albumin. Then, washed using PBS followed by incubated with primary antibody, and subsequently treated with an appropriate secondary antibody. The samples were then incubated in the dark at room temperature for 1 h. After washing with PBS, 4′, 4 6‐diamidino‐2‐phenylindole (DAPI; Sigma‐Aldrich) staining for DNA was performed, and the samples were mounted and observed using an X‐Light V3 (Crest Optics) confocal microscopy. For IHC observation, lung cancer tissues were fixed with 4% paraformaldehyde, dehydrated, and embedded in paraffin. The sections were heated to expose the epitopes with a microwave oven at 95°C for 25 min and then blocked with goat serum. After sequential incubations with the primary and secondary antibodies, the sections were stained using 3,3′‐diaminobenzidine (DAB) and then counterstained with hematoxylin. Images were obtained using a BX63 light microscope (Olympus). In this study, the IF and IHC were conducted by Gene Pool, Co., Ltd, Beijing, China.

### 4.9 Flow cytometry

Cell cycle and cell apoptosis were detected by flow cytometry. For the analysis of the cell cycle, cells were seeded and exposed to radiation at indicated dosages and timepoints. Cells were rinsed, trypsinized, and then centrifuged at 1000 rpm at 4°C for 5 min, and pellets were suspended in 500 mL of PBS containing propidium iodide (PI, 100 mg/mL) and ribonuclease (10 mg/mL) for 30 min at room temperature. Then detection was done using the NovoCyte 2060R flow cytometer (ACEA Biosciences Inc.). The G0–G1, S, and G2–M phases of the cell cycle were gated for quantification.

For the analysis of apoptosis, the Annexin V‐FITC Apoptosis Detection Kit (Beyotime) was used to stain cells, and the detection steps were followed according to the manufacturer's instructions. Then detection was done using the NovoCyte 2060R flow cytometer (ACEA Biosciences Inc.).

### 4.10 Cell counting kit‐8 (CCK8) assay

For the investigation of cell proliferation postradiation or post‐indicated interventions, CCK‐8 (Sungene Biotech) kits were purchased, and the detection steps followed the instructions. Briefly, the cells were seeded, radiated, and added to CCK‐8 solution, and the absorbance (OD) was detected using a microplate reader at 450 nm. Detection was repeated at least three times; average OD results were calculated for further statistic assay.

### 4.11 Western blotting (WB)

For Western blotting, lung tissue samples or cells were lysed in cell lysis buffer for extracting total protein, maintained under constant agitation for 30 min at 4°C. Nuclear and cytoplasmic proteins of the cells were isolated using a Nuclear and Cytoplasmic Protein Extraction Kit (Beyotime). The steps for WB assay have been followed by previous reports, and this method has been conducted in our lab for many published studies. SDS‐polyacrylamide gels transferred onto PVDF membranes (Roche) were purchased, and the relative antibodies were purchased from different companies. The Mini‐Trans Blot equipment was purchased from Bio‐Rad, USA to do WB Signals and were visualized using an Image Quant LAS4010 (GE Healthcare Bio‐Sciences). In addition, the gray assay was performed using ImageJ software.

### 4.12 JC‐1 assay

JC‐1 assay was used to detect the mitochondrial membrane potential (∆*ψm*). Cells were incubated with 2 µmol/L JC‐1 dye (Thermo Fisher Scientific) in a complete medium for 15 min at 37°C. Over 30 visual fields from the cells were analyzed using a Confocal Quantitative Image Cytometer (Yokogawa).

### 4.13 Immunoprecipitation (IP)

IP was performed to detect the interaction between proteins. Total cellular protein extracts (500 µg) were incubated with 1 µg antibody at 4°C for 1 h, followed by incubation with 20 µL Protein G PLUS‐Agarose (Santa Cruz) overnight at 4°C. After centrifugation at 1000 g, the supernatant was discarded, and the resulting immunoprecipitate was washed three times with lysis buffer. The sample was then resuspended in 1× electrophoresis sample buffer (Beyotime). Finally, the extracted proteins were detected by Western blotting.

### 4.14 Transmission electron microscopy (TEM)

In this study, the samples were collected and shipped to Shiyanjia Lab (www.shiyanjia.com) for TEM. The lung tissues were split and then washed using cold 2.5% glutaraldehyde first and then washed by PBS twice. Following post‐fixed, the samples were washed again with PBS. After that, dehydration was done for further being incubated. A JEM‐2100F at 80 kV was used, and a side‐inserted BioScan camera was for acquired photos.

### 4.15 Quantitative real‐time PCR (qRT‐PCR)

qRT‐PCR was done according to our previous report. Briefly, TRIzol reagent (Jingcai Bio., Xi'an, Shanxi, China) was used to extract RNA from cells or tissues. SYBR green dye (TB Green Premix Ex Taq II) was for quantification. Cycle program was as follows, 95°C for 10 min, 95°C for 15 s, and 60°C for 1 min, for 40 cycles. The detection equipment was CFX96 Touch apparatus (Bio‐Rad, USA). The relative expression was calculated using the 2‐ΔΔCT method.

The primers, TXNL4B: 5′‐CTGCCCAAGCTGACTAGCAA‐3′,

and reverse, 5′‐CTAGCTGCAGACAGACAGGA‐3′,

PRP3: 5′‐GAGAATGCGAAGGAACAAGC‐3′

and reverse, 5′‐AGTCTTGCCGCTGTAGGTAA‐3′;

GAPDH: 5′‐ACATCGCTCAGACACCATG‐3′

and reverse, 5′‐TGTAGTTGAGGTCAATGAAGGG‐3′.

### 4.16 Mice experiments (xenograft assay and radiation exposure)

Five‐week‐old female BALB/c nu/nu mice were purchased from Hunan SJA Laboratory Animal Company. In total, 5 × 10^6^ A549 cells expressing NT shRNA or shTXNL4B were subcutaneously injected into the dorsal lateral flank of nude mice to generate lung tumors. When the tumor volumes reached 200 mm^3^, the tumor‐bearing mice received a single 6 Gy dose of radiation to the tumor area, and the rest of their body was shielded using a lead plate. Thirty‐five days later, tumors were collected from three mice from each of the shTXNL4B‐ or NT shRNA‐expressing groups. Tumor volumes were measured using a ruler graduated in centimetres. Tumor volumes were calculated as follows: *V* (mm^3^) = (*a* × *b* ^2^)/2, where “*a*” refers to the longest diameter, and “*b*” refers to the shortest diameter. All procedures were performed in accordance with protocols approved by the Animal Welfare and Ethics Committee of the Central South University (2020sydw0110).

For exposure experiment, male C57BL/6 mice at 6 weeks of age from the Hunan SJA Laboratory Animal Co., LTD, China were divided into three groups, control group, 16 Gy on lung part group (Radiation), and 16 Gy along with shTXNL4B prior treatment group (Radiation+shTXNL4B). Lungs were collected on day 7 after exposure. HE staining, TEM observation, and Western blotting were used for the detection of relative values.

### 4.17 LC–MS/MS analysis

Samples were collected and sent for LC–MS/MS analysis by BiotechPack Scientific., Ltd. China. The details of condition selection and parameters introduction are listed in the Supporting Information section.

### 4.18 Online available databases

TXNL4B expression in various cancers was evaluated using the TCGA database (https://cistrome.shinyapps.io/timer/). The GEPIA database (http://gepia.cancer‐pku.cn/index.html) was used to analyze RNA‐seq data from 8587 normal and 9736 tumor tissue samples from the TCGA and GTEx projects. Human Protein Atlas (HPA, http://www.proteinatlas.org) database includes tissue, cell, and pathology atlases. UCSC (http://genome.ucsc.edu/) and Uniprot (https://www.uniprot.org/) databases were used to obtain the potential gene sequences and 3D protein structures. KEGG database was used to present the spliceosome procedure (https://www.kegg.jp/). GENEMANIA: http://genemania.org/.BioGRID: https://thebiogrid.org/. MEME (Motif‐based sequence analysis tools): https://meme‐suite.org/meme/.

### 4.19 Alternative splicing assay

Alternative splicing assay was conducted by Oebiotech, Co., Ltd, Shanghai, China. The assembled transcripts were compared with the transcripts annotated by the reference sequence using A S Profile Software.^50^, ^51^ The inclusions of new transcripts were identified as follows: (i) more than 200 bp away from the existing annotation transcript; (ii) the length was no less than 180 bp. The details of experiment steps are listed in the Supporting Information section.

### 4.20 Statistical analyses

All data are reported as means ± standard deviation, and a *p*‐value <0.05 was considered statistically significant (Student's *t*‐test). A fold change ≥2.0 and *p*‐value <0.05 indicated differential mRNA expression. Unpaired numerical data were compared using the unpaired *t*‐test (two groups) or analysis of variance (more than two groups). Statistical analyses were performed using SPSS for Windows software (ver. 22.0; SPSS Inc., Chicago, IL, USA). GraphPad Prism 9 software (GraphPad Software Inc.) was used for plotting the data.

## AUTHOR CONTRIBUTIONS

*Conceptualization; writing original draft manuscript; funding acquisition*: Ruixue Huang. *Formal analysis; and critically revised manuscript*: Le Zhang and Pingkun Zhou. *Investigation; cell and animal experiments*: Liang Xiao, Zhao Ju, Jing Xiang, Yin Wang, Long Yang, Ridan Lei, Yunfeng Nie, and Yan He. *Methodology; collection of patients samples; formal analysis*: Long Yang, Yunfeng Nie, and Yun He. *Investigation and critically polished the English editing*: Justyna Miszczyk. All the authors read and approved the manuscript.

## CONFLICT OF INTEREST STATEMENT

The authors declare no conflict of interests.

## ETHICS STATEMENT AND CONSENT TO PARTICIPATE

All included patients provided informed consent, and the Ethics Committee of the School of Public Health, Central South University approved this study (Approved No. XYGW‐2018‐08). The animal study was approved by the Animal Care and Use Committee at the Central South University (2020sydw0110).

## Supporting information

Supporting Information

## Data Availability

All data needed to evaluate the conclusions in the paper are present in the paper and/or the Supporting Information. Additional data related to this paper may be requested from the authors.
