# Supplementary material for: TXNL4B regulates radioresistance by controlling the PRP3‐mediated alternative splicing of FANCI
Source: MedComm (2020). 2023 May 7;4(3):e258. doi: 10.1002/mco2.258 (PMC10165318; doi:10.1002/mco2.258)
Supplement: Supplementary file 1 — Supporting Information [file MCO2-4-e258-s001.docx]

**TXNL4B regulates radioresistance by controlling the PRP3-mediated Alternative Splicing of FANCI**

Zhao Ju^1,2#^, Jing Xiang^1,2#^, Liang Xiao^3#^, Yan He^4#^, Le Zhang^5#^, Yin Wang^1^, Ridan Lei^1^, Yunfeng Nie^6^, Long Yang^6^, Justyna Miszczyk^7^, Pingkun Zhou^2^, Ruixue Huang^1*^

^1^Department of Occupational and Environmental Health, Xiangya School of Public Health, Central South University, Changsha, Hunan Province 410078, China.

^2^Department of Radiation Biology, Beijing Key Laboratory for Radiobiology, Beijing Institute of Radiation Medicine, AMMS, Beijing, China.

^3^Faculty of Naval Medicine, Naval Medical University (Second Military Medical University), Shanghai, 200433, China.

^4^Department of Ophthalmology, The Second Xiangya Hospital, Central South University, Changsha, Hunan, 410011, China, Hunan Clinical Research Center of Ophthalmic Disease.

^5^ Xiangya Hospital, Central South University, Changsha, Hunan Province 410078, China.

^6^Hunan Prevention and Treatment Institute for Occupational Diseases Changsha, Hunan Province 410078, China.

^7^Department of Experimental Physics of Complex Systems, The H. Niewodniczański Institute of Nuclear Physics Polish Academy of Sciences, Kraków, Poland. Electronic address:

#Zhao Ju, Jing Xiang, Liang Xiao, Yan He and Le Zhang contributed equally for this study.

Correspondence:

*Ruixue Huang, Department of Occupational and Environmental Health, Xiangya School of Public Health, Central South University, Changsha, Hunan Province 410078, China Tel: 0086-731-84805460, [huangruixue@csu.edu.cn(RX](mailto:huangruixue@csu.edu.cn(RX) H)


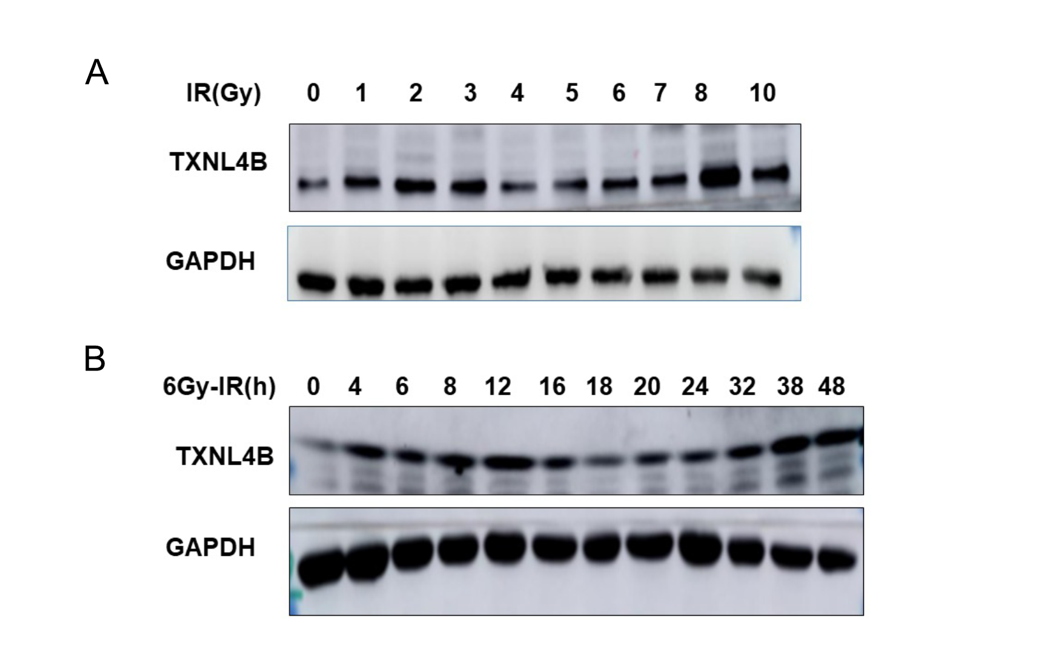


Supplementary Figure 1. A. TXNL4B expression increased in a dose-dependent relationship post radiation. B. TXNL4B expression increased in a time-dependent relationship post radiation. GAPDH serves as control.


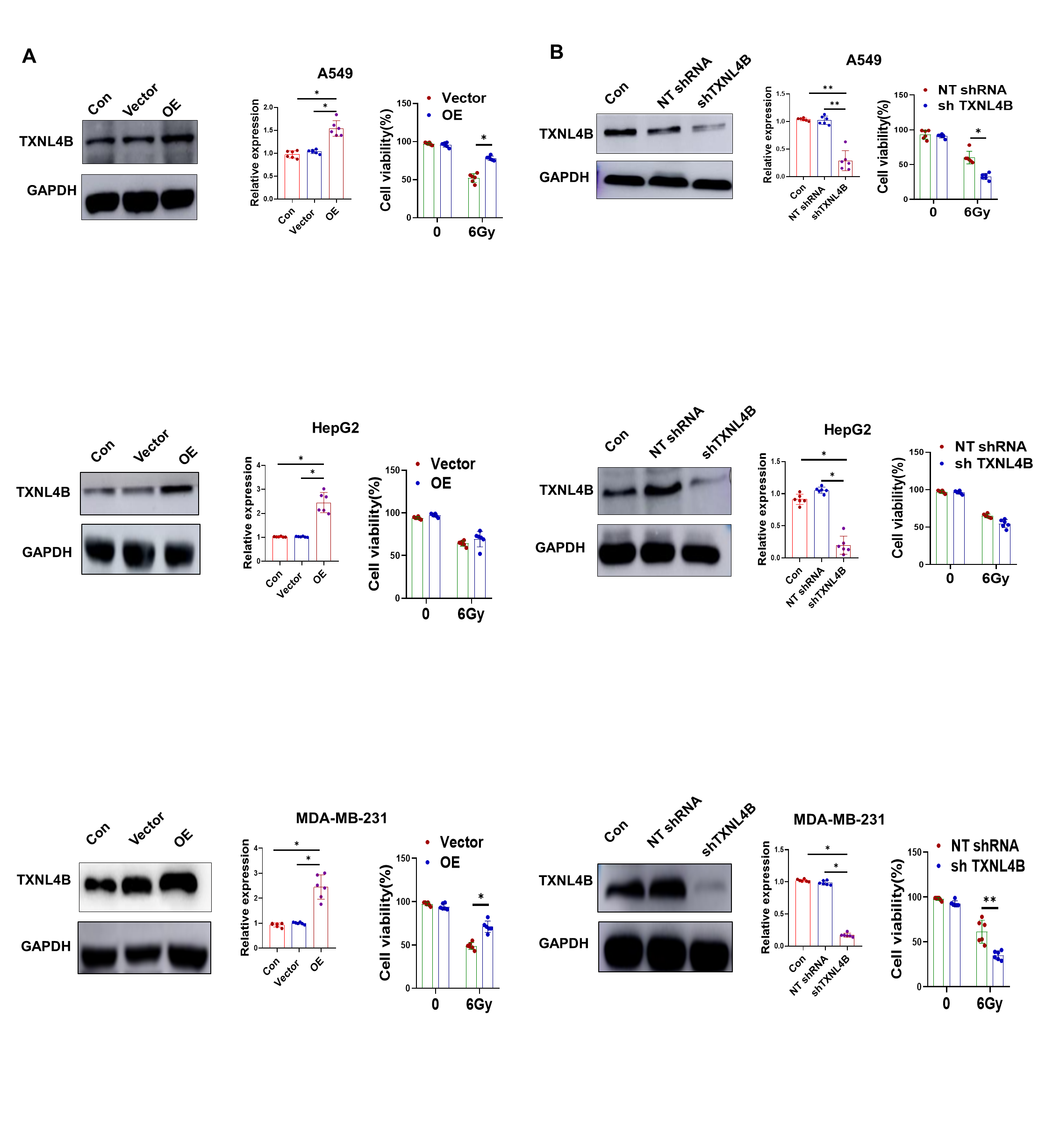


Supplementary Figure 2. TXNL4B expression is associated with radioresistance. A. The effect of TXNL4B overexpression in radioresistance estimated by cell viability assay in various cancer cell lines, A549, HepG2, and MDA-MB-231, respectively. B. The effect of TXNL4B knockdown in radioresistance estimated by cell viability assay in various cancer cell lines, A549, HepG2, and MDA-MB-231, respectively. GAPDH subjected to the loading control reference for Western blotting detection. Error bars represent the SD. Two-tailed, unpaired t test was used. **p*<0.05, ***p*<0.01.


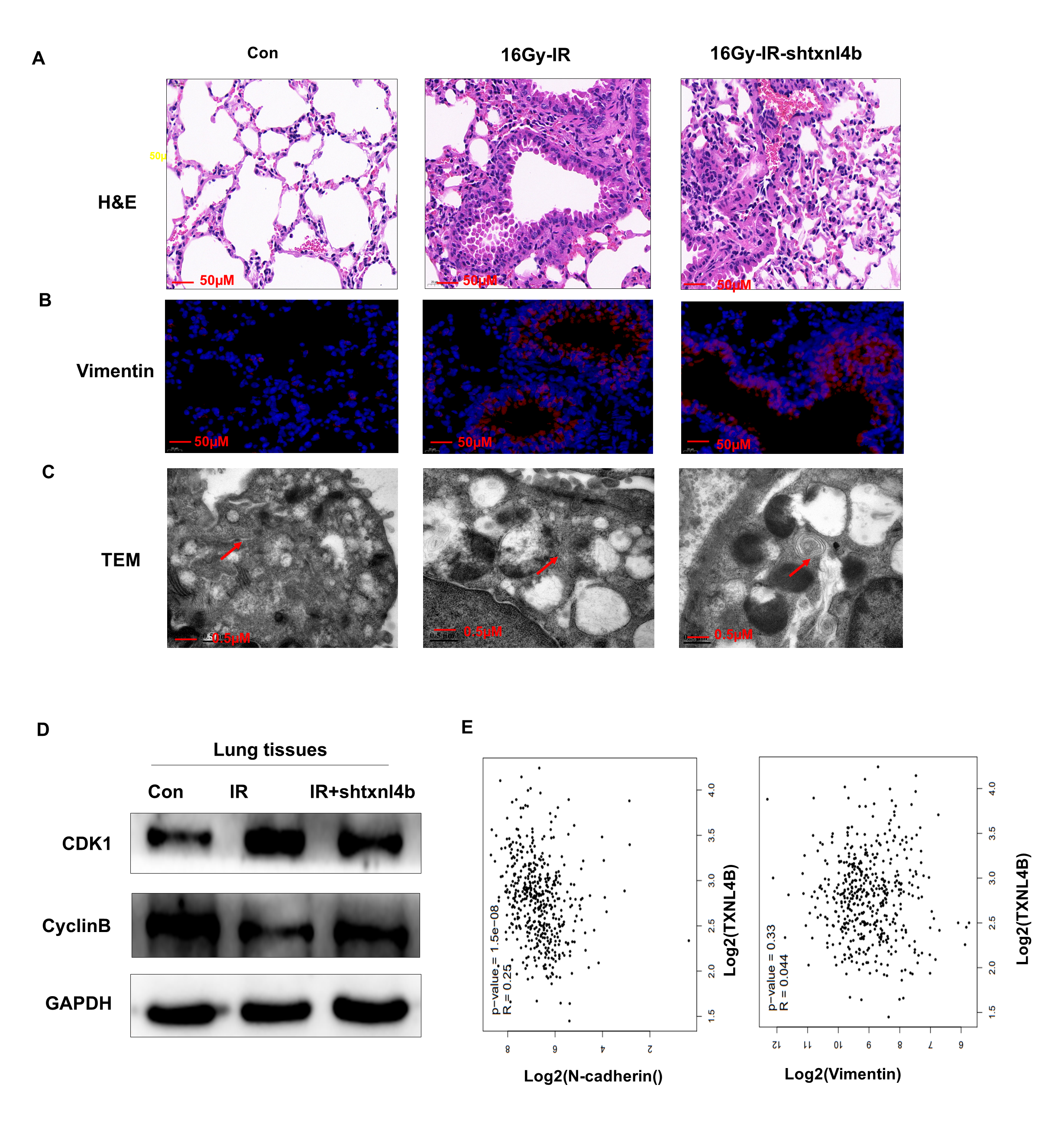


Supplementary Figure 3. A-C. Representative images of lung tissues, lung pathology, Vimentin expression and TEM lung structure among control group, 16Gy radiation group and 16Gy radiation with TXNL4B knockdown group, respectively ∆(n=6). Scale bar=50 µm for HE and IF, =0.5µm for TEM. D. protein expression within three groups. E. Correlation of TXNL4B expression and E-cadherin and Vimentin in lung cancer tissues based on the RNA-seq results from GEPIA website(<http://gepia.cancer-pku.cn/index.html>).


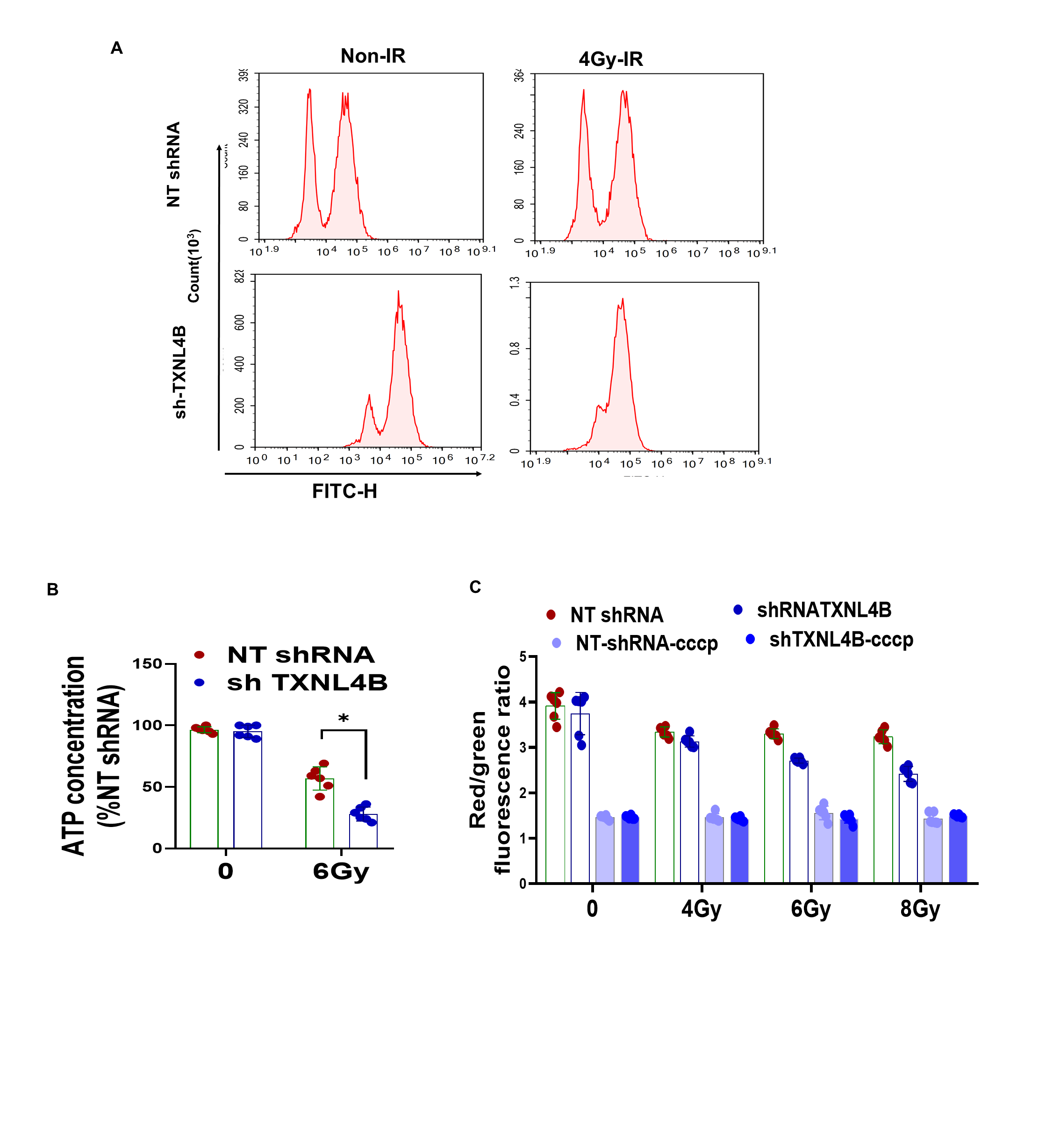


Supplementary Figure 4. A. The levels of reactive oxygen species (ROS) in NT-shRNA-A549 and shTXNL4B-A549 cells post 4Gy radiation were detected using Flow cytometry. B. ATP concentrations in NT-shRNA-A549 and shTXNL4B-A549 cells were detected using an ATP assay kit 4 h post 6Gy radiation. C. Carbony cyanide m-chlorophenyl hydrazine (CCCP) was used to gain positive control for loss of ∆ᴪm. Cells were treated with the doses of 4, 6 and 8Gy as indicated. Scale bar=50µm. Two-tailed, unpaired t test. Data are means ± SD from three independent experiments. **p*< 0.05, ***p*< 0.01.


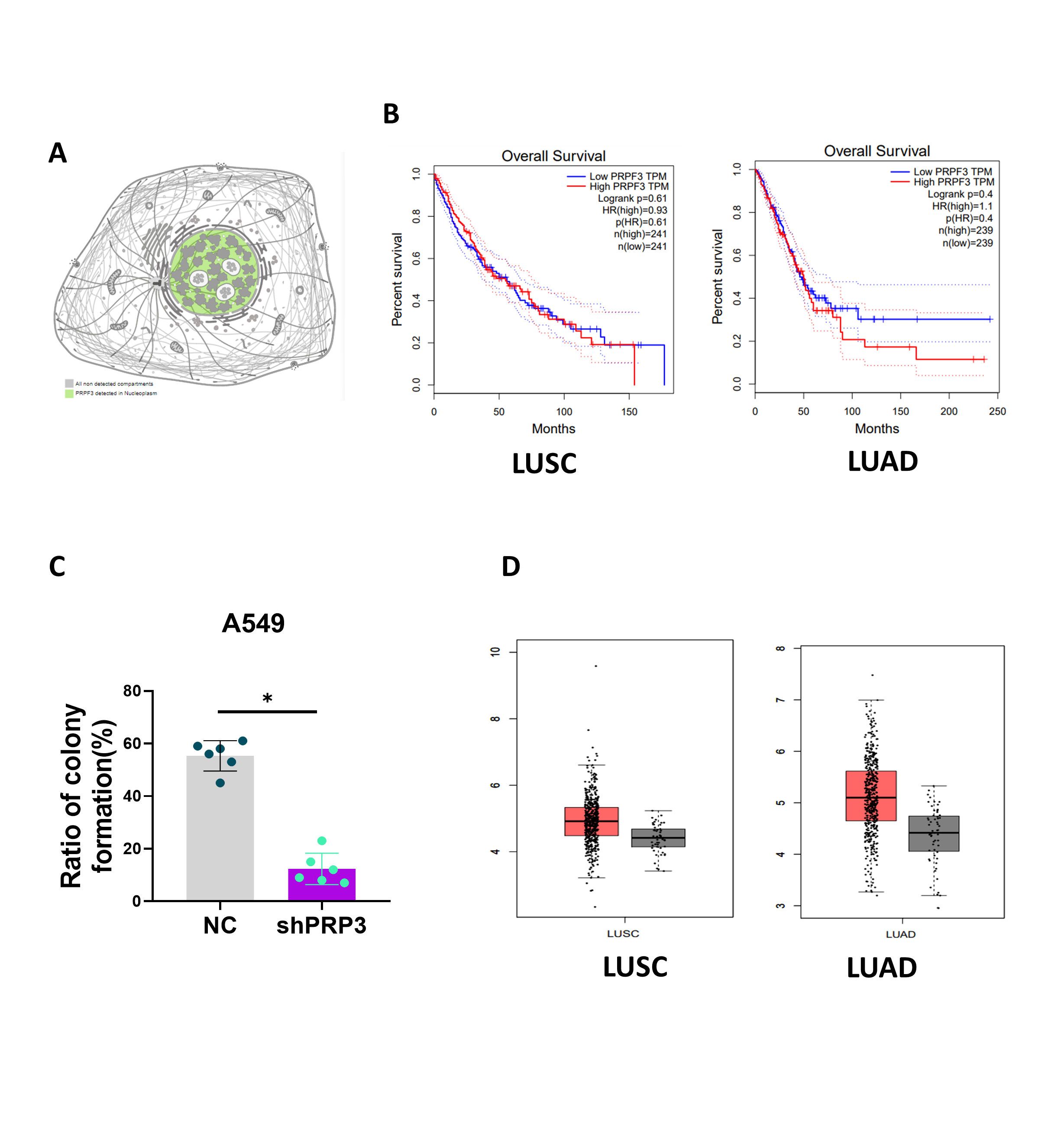


Supplementary Figure 5. A. PRP3 cell location (GEPIA website (<http://gepia.cancer-pku.cn/index.html>). B. Overall survival of patients of LUSC and LUAD with low versus high PRP3 expression, respectively. C. Ratio of colony formation (%) between PRP3-wt and PRP3-KD cells. D. The expression of PRP3 in normal lung tissues(N) and lung cancer(LC) tissues (<http://gepia.cancer-pku.cn/index.html>). Error bars represent the SD (Standard error). Data are means ± SD from three independent experiments. Two-tailed, unpaired t test was used. **p*<0.05, ***p*<0.01. LUSC: lung squamous cell carcinoma, LUAD: Lung adenocarcinoma.


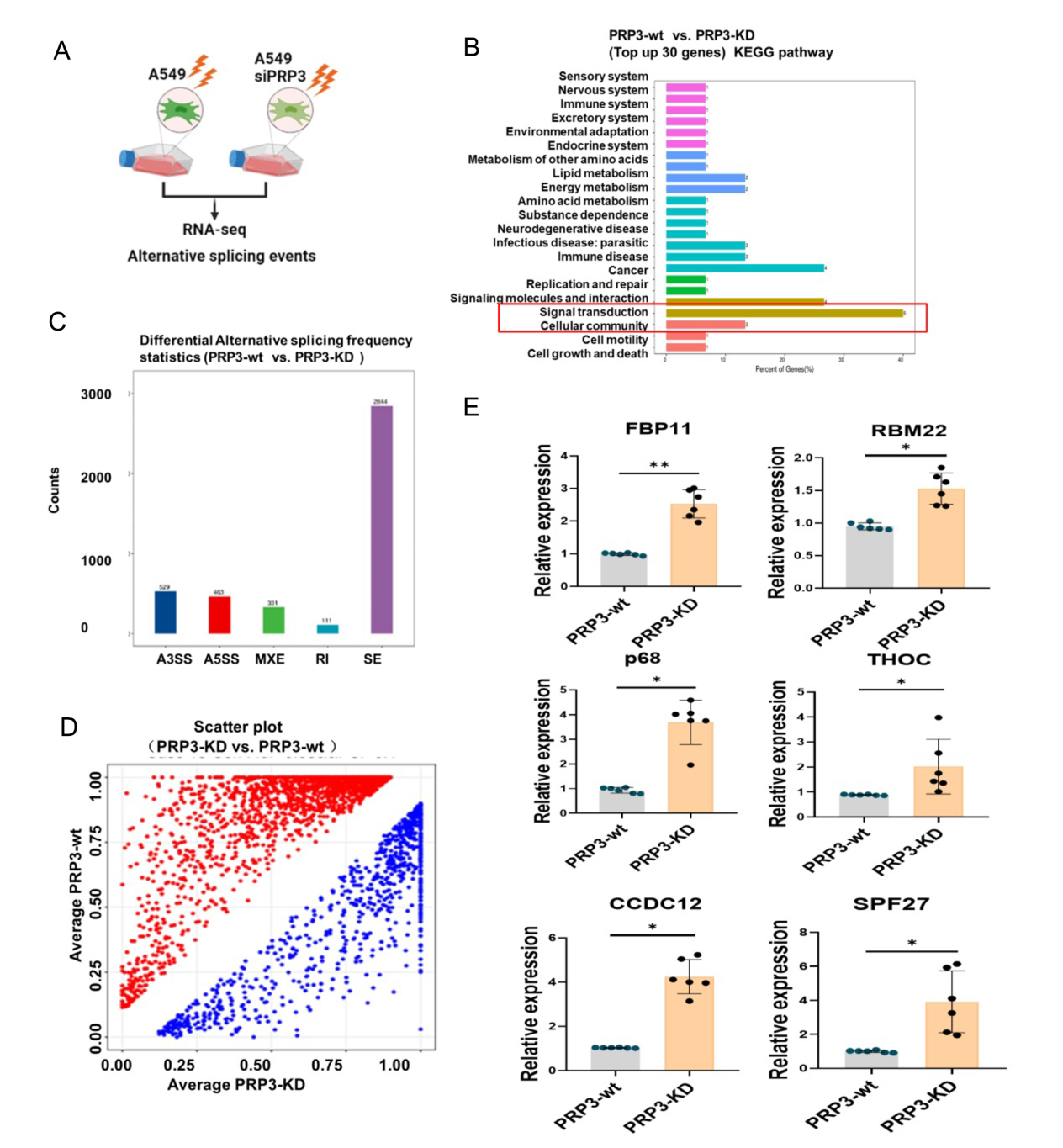


Supplementary Figure 6. A. Schedule of RNA-seq and alternative splicing events design. B. KEGG analysis of top 30 genes with alternative splicing between PRP3-wt and PRP3-KD cells post 6Gy radiation. C. 5 types of alternative splicing events identified by RNA-seq analysis of A549 cells after transfection with the indicated siRNAs after 6Gy radiation. D. Scatter plot of alternative splicing events between PRP3-wt and PRP3-KD cells post 6Gy radiation. E. Validation of indicated relative expression of genes between PRP3-wt and PRP3-KD cells post 6Gy radiation. Two-tailed, unpaired t test. Data are means ± SD from three independent experiments. **p*< 0.05, ***p*< 0.01.


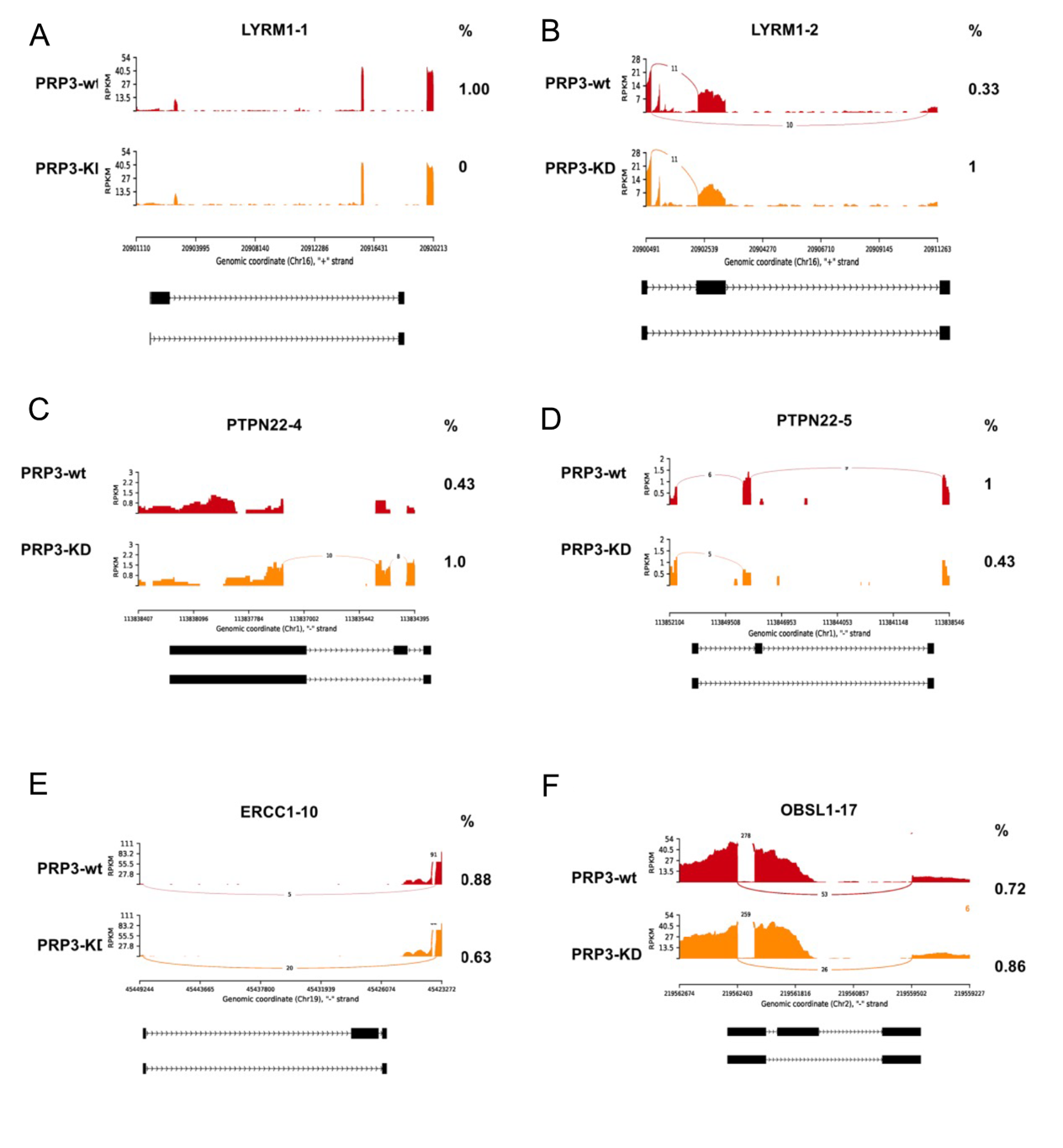


Supplementary Figure 7. A sashimi plot displaying the major splice junctions at LYRM1-1(A), LYRM1-2(B), PTPN22-4(C), PTPN22-5(D), ERCC1-10(E) and OBSL1-17(F) in the PRP3-KD cells and PRP3-wt cells post 6Gy.


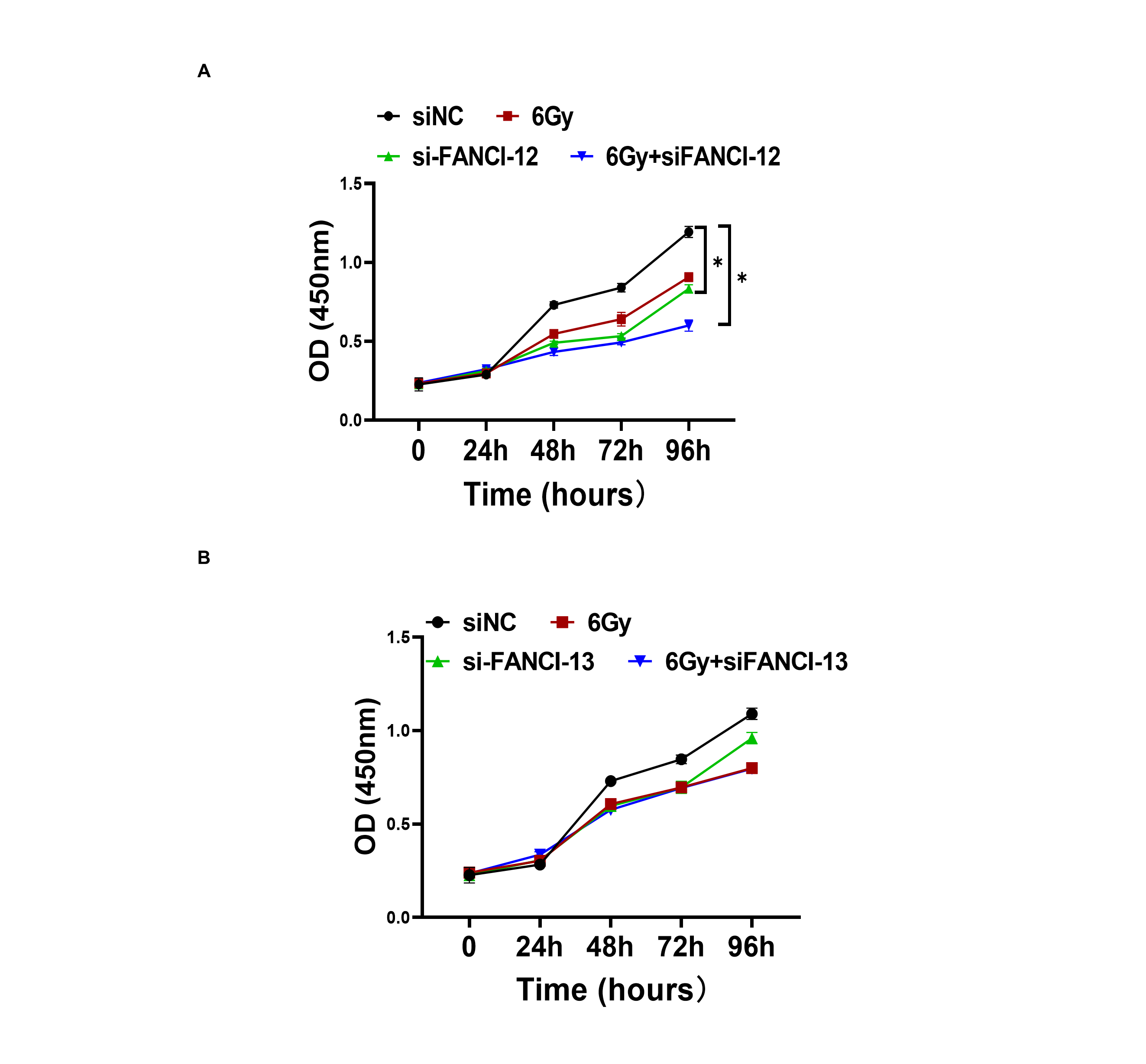


Supplementary Figure 8. A. Quantification of OD value in A549 cells after transfected with indicated siRNAs post 6Gy radiation at indicated timepoints. B. quantification of OD value in A549 cells after transfected with indicated siRNAs post 6Gy radiation at indicated timepoints. Error bars represent the SD (Standard error). Data are means ± SD from three independent experiments. Two-tailed, unpaired t test was used. **p*<0.05, ***p*<0.01.


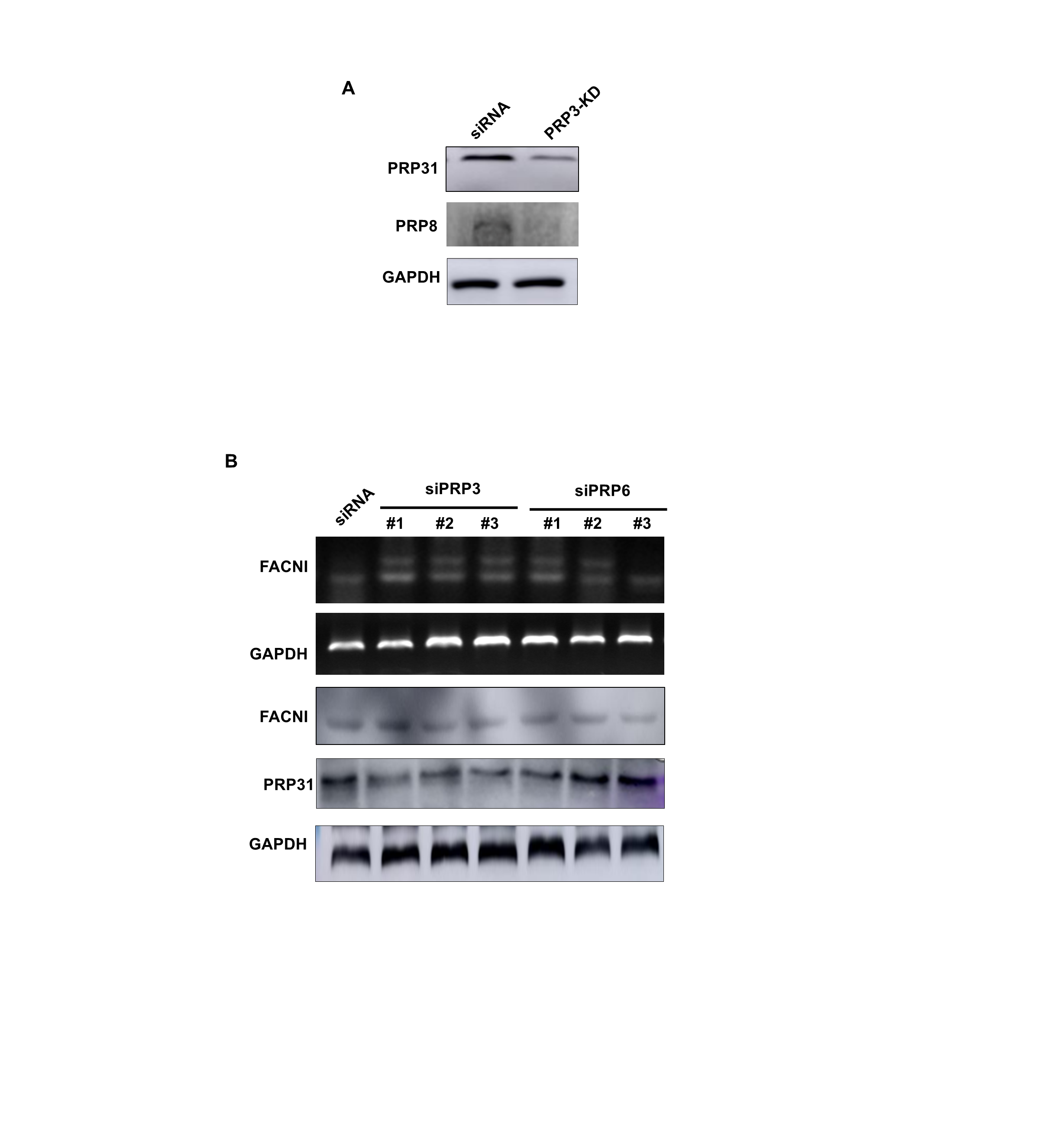


Supplementary Figure 9. A. PRP6 expression between PRP3-wt and PRP3-KD cells. B. RT-PCR and Western blotting analysis of PRP3, PRP6 and FANCI expression after transfected with indicated siRNAs. GAPDH subjected to the loading control reference for Western blotting detection.

Supplementary table 1 | Top 30 differential expression of genes between PRP3-wt and PRP3-KD cells post 6Gy radiation

| **Up (**PRP3-KD vs. PRP3-wt) | | | **Down (**PRP3-KD vs. PRP3-wt) | | | | |
| --- | --- | --- | --- | --- | --- | --- | --- |
| **Gene** | **Log2 fold change** | **P value** |  |  | **Gene** | **Log2 fold change** | **P value** |
| CBSL | 5.390245 | 3.42E-25 |  |  | LTB | -1.00091 | 6.73E-06 |
| FBP11 | 3.08985 | 0.039615 |  |  | HNRNPD | -1.00607 | 0.035198 |
| MATN1 | 3.08985 | 0.039615 |  |  | TMSB15B | -1.03943 | 0.037609 |
| CA150 | 2.727279 | 0.001578 |  |  | SPOCD1 | -1.0416 | 0.03217 |
| RBM22 | 2.334962 | 0.013285 |  |  | U2AF | -1.0426 | 0.002516 |
| SULT1A4 | 2.077466 | 8.99E-06 |  |  | TMIE | -1.0497 | 0.015204 |
| OR51B4 | 2.035402 | 0.045443 |  |  | GRIK5 | -1.08008 | 0.04701 |
| LAMC3 | 1.978818 | 0.004755 |  |  | HBQ1 | -1.08008 | 0.034139 |
| SLX1B | 1.933221 | 1.76E-08 |  |  | SLC34A3 | -1.08008 | 0.04701 |
| OCLM | 1.919925 | 0.030397 |  |  | SIK1 | -1.09869 | 1.07E-05 |
| SGK2 | 1.919925 | 0.014271 |  |  | PHKG1 | -1.21758 | 0.02077 |
| RGPD5 | 1.89038 | 2.60E-10 |  |  | KRT17 | -1.25 | 0.033066 |
| MAP1A | 1.75 | 0.003338 |  |  | LOC105370687 | -1.25 | 0.033066 |
| JAKMIP1 | 1.697532 | 0.013513 |  |  | FBP1 | -1.402 | 0.024366 |
| GRPR | 1.685459 | 0.042118 |  |  | ZNF556 | -1.66504 | 0.030692 |
| CYP19A1 | 1.443487 | 0.03347 |  |  | FAM166A | -1.72393 | 0.008082 |
| SPF27 | 1.289158 | 0.00141 |  |  | CBLC | -1.74304 | 0.021143 |
| SALL2 | 1.277477 | 0.009435 |  |  | LOC112268184 | -1.74304 | 0.021143 |
| P68 | 1.267848 | 0.033791 |  |  | CYP26B1 | -1.75815 | 0.034806 |
| THOC | 1.241853 | 0.049568 |  |  | LSMEM2 | -1.78052 | 0.005616 |
| GTF2IRD2 | 1.241853 | 0.012253 |  |  | POPDC2 | -1.98697 | 0.02588 |
| IL7R | 1.241853 | 0.049568 |  |  | LIMS3 | -2.08008 | 0.001297 |
| RHBDL3 | 1.241853 | 0.019194 |  |  | WNT4 | -2.08008 | 0.017018 |
| CCDC12 | 1.205327 | 0.015853 |  |  | MXRA8 | -2.402 | 0.01127 |
| CPM | 1.200032 | 0.024969 |  |  | TBC1D3D | -2.53951 | 0.028476 |
| C6orf99 | 1.192943 | 0.039992 |  |  | RGPD6 | -2.58258 | 1.18E-08 |
| GTPBP6 | 1.190778 | 9.49E-05 |  |  | C11orf98 | -2.66504 | 0.017509 |
| CASKIN1 | 1.182959 | 0.022581 |  |  | TMEM190 | -3.08008 | 0.045532 |
| MROH7 | 1.156964 | 0.032332 |  |  | KCNJ9 | -3.123 | 0.010119 |
| FLT1 | 1.126375 | 0.046552 |  |  | LRRC66 | -3.2875 | 0.035788 |

Supplementary Table 2| The differential transcripts of genes affected by five types of alternative splicing

| **A3SS** | **A5SS** | **MXE** | **RI** | **SE** |
| --- | --- | --- | --- | --- |
| ARL6IP4-3 | AGO3-19 | ASAH2-16 | ATG4B-16 | ARL17B-20 |
| ARMCX5-9 | BCAS4-3 | CDKL3-17 | BORCS7-8 | CAMK2D-9 |
| ATL2-8 | CAMK2D-1 | COP1-12 | C18orf21-9 | CLIP4-18 |
| E4F1-14 | CEP128-11 | CRIM1-9 | CAPN15-20 | CLIP4-19 |
| GOLGA7-19 | CPSF1-5 | DTYMK-2 | CBS-13 | ERCC1-10 |
| HARS2-10 | CTNND1-20 | FANCI-1 | DAB21P-12 | FANCI-12 |
| IDUA-12 | E4F1-12 | GPR137-11 | DVL3-18 | FANCI-13 |
| MTF2-16 | GTF3C1-9 | KLK10-18 | IDUA-4 | FHIT-8 |
| NKIRAS1-20 | KCNK5-14 | LRRC14-20 | KRIT1-15 | IFRD2-7 |
| PAICS-2 | LPXN-10 | LYPD6B-3 | MCMDC2-10 | LYRM1-1 |
| POC1B-GALNT4-1 | MOK-2 | MYD88-6 | PPOX-14 | LYRM1-2 |
| PPFIA1-4 | NCEH1-13 | NEPRO-15 | SLC9A7-3 | LYRM1-3 |
| RABL2B-15 | OARD1-4 | PDZD2-10 | SLTM-11 | MOK-14 |
| RINT1-6 | SAXO2-17 | PPFIA1-4 | TDRKH-6 | MOK-15 |
| SAMD10-11 | STK32B-8 | PPFIA1-5 | UBE2D4-2 | MOK-16 |
| SMG6-17 | TANC2-16 | SLC38A1-19 | USP39-1 | OBSL1-17 |
| STRN4-7 | TIRAP-18 | STRN4-7 | WASHC2A-7 | POC1B-GALNT4-11 |
| TGFB1-5 | TMEM107-15 | TEX264-13 | ZBTB44-19 | PTPN22-4 |
| TRPM7-13 | VAMP7-6 | TMEM116-8 | ZNF7-5 | PTPN22-5 |
| ZFP69B-18 | VAMP7-7 | ZCCHC14-14 | ZNF226-17 | WRAP73-6 |

Supplementary Methods for LC-MS/MS condition.

Nanoflow UPLC: Easy-nLC1200 system (ThermoFisher Scientific, USA); Nanocolumn: 150 μm×15 cm in-house made column packed with a reversed-phase ReproSil-Pur C18-AQ resin (1.9 μm, 100 Å, Dr. Maisch GmbH, Germany); Loaded sample volume: 5 μL; Mobile phase: A: 0.1% formic acid in water; B: 20% 0.1% formic acid in water-80% acetonitrile. Total flow rate : 600 nL/min; LC linear gradient: from 4% to 8% B for 2 min, from 8% to 28 % B for 43 min, from 28 % to 40% B for 10 min, from 40% to 95% B for 1 min and from 95% to 95% B for 10 min. Mass spectrometry,Q Exactive™ Hybrid Quadrupole-Orbitrap™ Mass Spectrometer Thermo Fisher Scientific, USA), Spray voltage: 2.2 kV; Capillary temperature: 270°C; MS resolution: 70000 at 400 m/z; MS precursor m/z range: 300.0-1800.0; MS/MS parameters: product ion scan range from m/z 200; Activation Type: HCD; Normalized Coll. Energy: 28.0; Activation Time: 66.000; Data dependent MS/MS: up to top 20 most intense peptide ions from the preview scan in the Orbitrap. The raw MS files were analyzed and searched against protein database based on the species of the samples using MaxQuant (1.6.2.10). The parameters were set as follows: the protein modifications were carbamidomethylation (C) (fixed), oxidation (M) (variable), Acetyl (Protein N-term) (variable); the enzyme specificity was set to trypsin; the maximum missed cleavages were set to 2; the precursor ion mass tolerance was set to 20 ppm, and MS/MS tolerance was 20 ppm. Only high confident identified peptides were chosen for downstream protein identification analysis.

Supplementary Methods for Alternative splicing assay.

We used the event-based tool rMATS (turbo v.4.1.095) to quantify both annotated and novel splice junctions in the PRP3-wt (n = 3) and PRP3-KD (n=3) post radiation, and to assess differential splicing between the two groups. The rMATS program identified five types of splice event: alternative 3’ spike site (A3SS), alternative 5’ splice site (A5SS), skipped exon (SE; also called cassette exons), intron retention (IR). And mutually exclusive exons (MXE), by default including all events with, at least one read supporting the exon inclusion form and the exon skipping. Raising this threshold caused the number of splice events to drop steeply, as did adjusting the minimum number of samples with at least one read supporting the inclusion or exclusion form. To be considered a significantly differentially spliced event, we required splice sites to have adjusted p-values below 0.05 and a △PSI value of >5%, a cut-off that was chosen based on other event-based differential splicing analyses. Expected number of dif­ferentially spliced sites was calculated as the product of the subtotal of each splice type and the proportion of total splice events that were significant.
